# Explainable artificial intelligence (XAI) for exploring spatial variability of lung and bronchus cancer (LBC) mortality rates in the contiguous USA

**DOI:** 10.1038/s41598-021-03198-8

**Published:** 2021-12-16

**Authors:** Zia U. Ahmed, Kang Sun, Michael Shelly, Lina Mu

**Affiliations:** 1 Research and Education in Energy, Environment and Water (RENEW) Institute, University at Buffalo, 108 Cooke Hall, Buffalo, NY 14260 USA; 2 Department of Civil, Structural and Environmental Engineering, University at Buffalo, 230 Jarvis Hall, Buffalo, NY 14260 USA; 3 Department of Epidemiology and Environmental Health, University at Buffalo, 273A Farber Hall, Buffalo, NY 14214 USA

**Keywords:** Risk factors, Machine learning

## Abstract

Machine learning (ML) has demonstrated promise in predicting mortality; however, understanding spatial variation in risk factor contributions to mortality rate requires explainability. We applied explainable artificial intelligence (XAI) on a stack-ensemble machine learning model framework to explore and visualize the spatial distribution of the contributions of known risk factors to lung and bronchus cancer (LBC) mortality rates in the conterminous United States. We used five base-learners—generalized linear model (GLM), random forest (RF), Gradient boosting machine (GBM), extreme Gradient boosting machine (XGBoost), and Deep Neural Network (DNN) for developing stack-ensemble models. Then we applied several model-agnostic approaches to interpret and visualize the stack ensemble model's output in global and local scales (at the county level). The stack ensemble generally performs better than all the base learners and three spatial regression models. A permutation-based feature importance technique ranked smoking prevalence as the most important predictor, followed by poverty and elevation. However, the impact of these risk factors on LBC mortality rates varies spatially. This is the first study to use ensemble machine learning with explainable algorithms to explore and visualize the spatial heterogeneity of the relationships between LBC mortality and risk factors in the contiguous USA.

## Introduction

Lung and bronchus cancer (LBC) is one of the most common causes of cancer death globally, accounting for 11.6% of all cancer deaths in 2018^1^. It contributes substantially to healthcare costs and the health burden globally^2^ and is an insistent public health concern due to its low survival rate^3^. In the USA, the LBC mortality rate declined by 48% from 1989 to 2016^3^, but it remains the top cause of cancer-related death^4^. An estimated 142,670 Americans were expected to die from lung cancer in 2019, approximately 23 percent of all cancer deaths^3^. LBC mortality rates vary substantially between and within states in the US^3,5^. This variation has been mainly linked to variation in smoking prevalence^6^. Yet, causes of lung cancer mortality are more complex^7^ and are also linked with air pollution^8^, and socioeconomic conditions^3,9^. Some of these risk factors have not been previously included in the modeling of predicting the LBC mortality rate^7,8,10–13^.

Several statistical methods and tools have been developed to analyze and report cancer incidence and mortality statistics in the USA, including the Poisson-gamma model, the multivariate conditional autoregressive model, and Bayesian inference^14^. The state‐space method (SSM)^15^ and autoregressive quadratic time trend model^16^ are primarily used to estimate the total number of cancer deaths expected to occur in a given period. Numerous studies have applied Geographically weighted (GW) models to explore the geographic relationship between risk factors and the LBC mortality rate^7,8,17–19^. However, a traditional linear model may fail to capture complex interactions and non-linear relationships between LBC mortality and risk factors. The increasing availability of data and machine learning (ML) models present an opportunity to predict and identify the factors contributing to the LBC mortality rate and help develop a strategy for targeting areas for the management of treatment. The machine learning approach has been recently applied to other health problems such as arrhythmia detection^20^, disease incidence^21^, the mortality rate^22,23^, and cancer survival prediction^24^. Recently, stacked generalization or stacking, or super learning, which introduces a meta-learner concept that combines multiple classifiers or regression models, has been used to improve predictive accuracy^25–27^. Some ML models are intrinsically capable of explaining knowledge about domain relationships in data, known as the interpretability of the ML models^28^. However, many ML models are "Black boxes," "meaning their internal logic and inner workings are hidden to the user and even experts cannot fully understand the rationale behind their predictions" (Carvalho et al., 2019). The lack of "transparency and accountability" of ML models can have some drawbacks when applied to healthcare, criminal justice, and other regulated domains for high-stakes decision-making^29^. Higher interpretable models are easier to understand and explain the contribution of features in predictions^30^.

Although interpretability and explainability are often used interchangeably in ML, "explainable AI (XAI)" typically refers to post hoc analyses and techniques used to understand a previously trained "black-box models" or its predictions^31,32^. In particular, the Locally Interpretable Model-agnostic Explanations (LIME) technique is model agnostic proposed by Ribeiro et al. (2016), which can be used to interpret nearly any kind of machine learning models and their predictions^31^. The model-agnostic involves learning an interpretable model on the black box model's predictions, perturbing features, and seeing how the black box model reacts^33^ or both^34^. The LIME techniques have recently been used for explaining "black-box" predictions for a single observation or group of observations^35,36^. The "model agnostic greedy explanations of model predictions" or "break-down plot"^37^ can be used as an alternative to the well-known geographical weighted models^7,8,17–19^ to explore the spatial variability of local contribution of risk factors to the prediction. However, applying the model agnostic greedy explanations technique in the "black box" stack-ensemble model for explaining spatial heterogeneity in the relationship between county-level LBC mortality rate and risk factors has not been attempted.

First, we evaluated the performance of multiple machine learning (base learners) and spatial regression models for county-level LBC mortality rates prediction using many risk factors. Then we developed stacked ensemble models with these base learners to predict LBC mortality rates. Finally, we applied several model-agnostic interpretation methods to investigate the effects of several well-known risk factors on LBC mortality rates in the US, including permutation-based feature importance, partial dependence (PD), local-dependence (LD), and accumulated-local (AL) profiles. We also applied "model greedy agnostic explanations of model predictions" or "break-down plot" to explore and visualize the spatial distribution of the contributions of known risk factors to LBC mortality rates in the conterminous US. Several risk factors were used to train all models: county-level long-term average total cigarette smoking prevalence, poverty, health insurance, demography, biophysical factors (elevation, radon-zone, and urban–rural environment), and the satellite-derived annual average ambient atmospheric concentrations of particulate matter with a diameter of 2.5 microns or less (PM_2.5_), nitrogen dioxide (NO_2_), sulfur dioxide (SO_2_), and ozone.

## Material and methods

### Data

#### Lung and bronchus cancer (LBC) mortality rates by county

The county-level age-adjusted annual LBC mortality rates from 2013 to 2017 were obtained from the National Vital Statistics System at the National Center for Health Statistics of the Centers for Disease Control and Prevention^16,38,39^. The detailed extraction and age adjustment methods of mortality data are described elsewhere^40^. Due to data suppression for reliability and confidentiality, missing LBC mortality rate data in 348 counties in the contiguous USA counties were imputed with *missForest* package^41^ in R. The out-of-bag (OOB) imputation error (MSE) estimate was 35 per 100,000. Finally, we created  a data-frame of 3107 counties in the conterminous US. We did not include Shannon county in South Dakota due to a miss-match between the new and old FIPS codes, unique county identification numbers (Fig. 2).

#### Risk-factors

We assembled a comprehensive set of county-level risk factors (Table S2) to develop models to predict county-level LBC mortality rate in the contiguous USA. These data include variables relating to lifestyles, socio-economy, demography, air pollution, and physical environments.

##### Cigarette smoking prevalence

Data on age-adjusted cigarette smoking prevalence by county from 2008 to 2012 was obtained from the Institute for Health Metrics and Evaluation^42^, which derived the data from the results of the Behavioral Risk Factor Surveillance System (BRFSS) by using a logistic, hierarchical, mixed-effects regression model with spatial and temporal smoothing^43^. The BRFSS is a state-based random digit dial (RDD) telephone survey conducted annually in all states, the District of Columbia, and US territories. For the year 2008 to 2012 estimation, the root means squared error for male and female cigarette smoking was 1.9 for 100 sample size^43^. Data from 2013 to 2017 were obtained from County Health Ranking^44^, who also used BRFSS survey data to estimate county averages of age-adjusted cigarette smoking (%) prevalence. Before 2016, up to seven survey years of BRFSS data were aggregated to produce county estimates. The 2016 and 2017 data were obtained from single-year 2014 and 2015 BRFSS survey data, respectively. The average (2008–2017) smoking prevalence by county is shown in Fig. S1a.

##### Poverty rate

The data on the average (2012–2016) annual age-adjusted poverty data (% population below poverty level) by county are shown in Fig. S1b. The data were obtained from the US Census Bureau's Small Area Income and Poverty Estimates (SAIPE) program (US Cenus^45^. The county level observations from the American Community Survey (ACS) and census data were used to predict the number of people in poverty^46^. The ACS is an ongoing survey program conducted by the Census Bureau to provide vital population and housing information across the country^47^.

##### Uninsured percentage

Data on the portion of the population under age 65 without health insurance coverage from 2013 to 2017 (Fig. S1c) was obtained from Small Area Health Insurance Estimates (SAHIE) program^45^. The SAHIE program produces model-based health insurance coverage estimates for demographic groups within counties and states^48^.

##### Demography

County-level demography data such as white, non-Hispanic population (%,), black or African American, non-Hispanic population (%), Hispanic/Latino population (%), and population aged 65 and older (%) were obtained from the US Census^49^. We used the 5-year means (2013–2017) of these data in our study (Fig. S2a–d).

#### Air pollution

##### Particulate matter (PM_2.5_)

The county-level annual PM_2.5_ data were derived from the daily PM_2.5_ data set downloaded from the CDC data portal^50^. This county-level of 24-h average PM_2.5_ concentrations was generated by the US Environmental Protection Agency (EPA) using a Bayesian spatial downscaling fusion model^51^. For each county, annual PM_2.5_ from 2006 to 2016 was averaged to yield long-term yearly averages, which are mapped in Fig. S3a.

##### Nitrogen dioxide (NO_2_)

Population-weighted NO_2_ concentrations at 0.1° × 0.1° resolution were estimated using imagery from three satellite instruments, including the Global Ozone Monitoring Experiment (GOME), Scanning Imaging Absorption Spectrometer for Atmospheric Chartography (SCIAMACHY), and GOME-2 satellite in combination with the GEOS-Chem chemical transport model^52^. We resampled all raster data at a 2.5 km × 2.5 km grid size using Empirical Bayesian Kriging. We then averaged the results within each county for each year to yield a long-term annual average of NO_2_ that was mapped from 2003 to 2012 (Fig. S3b).

##### Sulfur dioxide (SO_2_)

Gridded (1-degree spatial resolution) annual, mean SO_2_ vertical column densities were obtained from time-series, multi-source SO_2_ emission retrievals, and satellite SO_2_ measurements from the Ozone Monitoring Instrument (OMI) on NASA's Aura satellite^53^. We resampled all raster data at a 2.5 km × 2.5 km grid size using Empirical Bayesian Kriging and then averaged the results within each county for the period from 2005 to 2015 (Fig. S3c).

##### Ozone

Annual county-level ozone data were derived from the Daily County-Level Ozone Concentrations downloaded from the CDC's data portal (CDC^54^, 2020). The daily data provide modeled predictions of ozone levels from the EPA's Downscaler model. The long-term average ozone concentration was generated from annual ozone data from 2006 to 2016 and mapped from 2007 to 2016 (Fig. S3d).

#### Biophysical factors

##### Radon zone

County-level radon zone data were downloaded from the EPA Radon zone interactive information site^55^. The radon zoning was done using indoor radon measurements, geology, aerial radioactivity, soil parameters, and foundation types. There are three radon zones differentiated by their predicted average indoor radon levels: Zone-1(> 4 pCi L^−1^), Zone-2 (2–4 pCi L^−1^), and Zone-3 (< 2 pCi L^−1^) (Fig. S4a).

##### Urban–rural counties

The data on the division of counties into urban or rural was drawn from the National Center of Health Statistics (NCHS) data system's Urban–Rural Classification Scheme for Counties^56^. All counties were classified into six classes based on the metropolitan statistical areas (MSA)^57^. We then reclassified the counties into four major classes: large central metro, large fringe metro, medium/small metro, and nonmetro (Fig. S4b).

##### Coal counties

We classified the counties into two classes (yes = coal produced, no = no coal production) according to the average coal production from 2006 to 2016 (Fig. S4c). We used data from the U**S** Energy Information Administration and the US Mine Safety and Health Administration's annual survey of coal production by US coal mining companies^58^. Data includes coal production, company and mine information, operation type, union status, labor hours, and employee numbers.

##### Elevation

We used elevation data from USGS^59^. Median elevation (m) for each county (Fig. S4d) was calculated.

### Analytical methods

**Figure 1:**
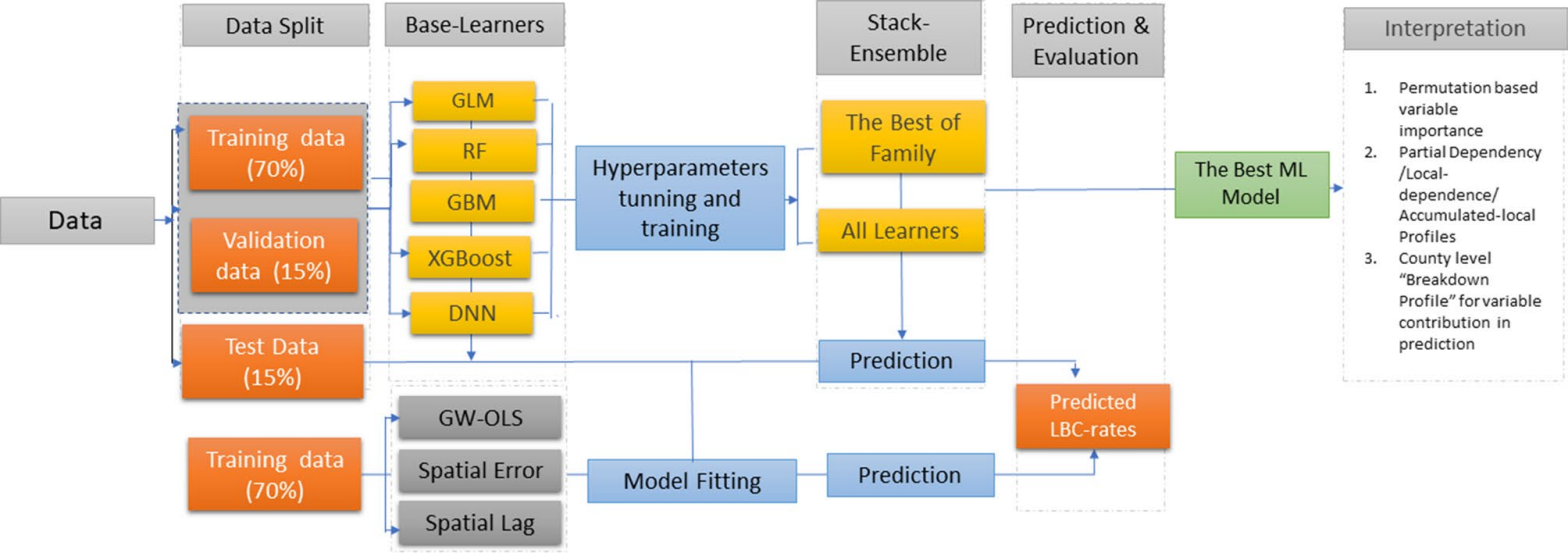
Steps used in meta-ensemble machine learning regression models for LBC morality rates prediction. GLM = generalized linear model, RF = random forest, GBM = Gradient boosting machine*,* XGBoost = extreme gradient boosting machine, DNN = Deep Neural Network; GW-OLS = Geographically Weighted OLS Regression (GW-OLS).

We developed stacked ensemble models from the output of five ML models to predict and explain the county-level LBC mortality using many risk factors (Fig. 1). We applied a series of model-agnostic interpretation methods to investigate the effects of several well-known risk factors on LBC mortality rates in the US. Three spatial regression models were used to evaluate the performance of the stack-ensemble model.

#### Exploratory data analysis

Before developing the machine learning model, we explored spatial autocorrelation and stratified spatial heterogeneity (SSH) of LBC mortality rates. Spatial autocorrelation assessment comprises statistics describing how a variable is autocorrelated through geographical space^60^. We used Getis-Ord Gi statistics^61^ to quantify spatial autocorrelation of LBC mortality rates by estimating the *z*-scores and *p*-values in each county. Larger statistically significant positive and negative z-scores indicate more intense clustering of high low values, respectively. We used ArcGIS Spatial Statistics Tools^62^ to estimate Getis-Ord Gi statistics for spatial autocorrelation. We also estimated bivariate Local Moran I (LMI) statistics to explore the degree of linear association between LBC mortality rates and risk factors at a given location and the average of another variable at neighboring areas (spatial lag).

Since our study area is vast, there is a possibility of high stratified spatial heterogeneity (SSH) which refers to a partition of a study area, where variables are homogeneous within each stratum but not between strata^63^. The q-statistic proposed by Wang et al.^63^ measures the degree of SSH in geographical space related to the ratio between the variance of a variable within the strata and the pooled variance of an entire study area. The value of the q-statistic range from 0 to 1, and it increases as the strength of the SSH increases. The calculated q-statistics for all risk factors used the "factor_detector" function of "geodetector" package^63^ in the R statistical computing environment^64^.

##### Training

**Figure 2:**
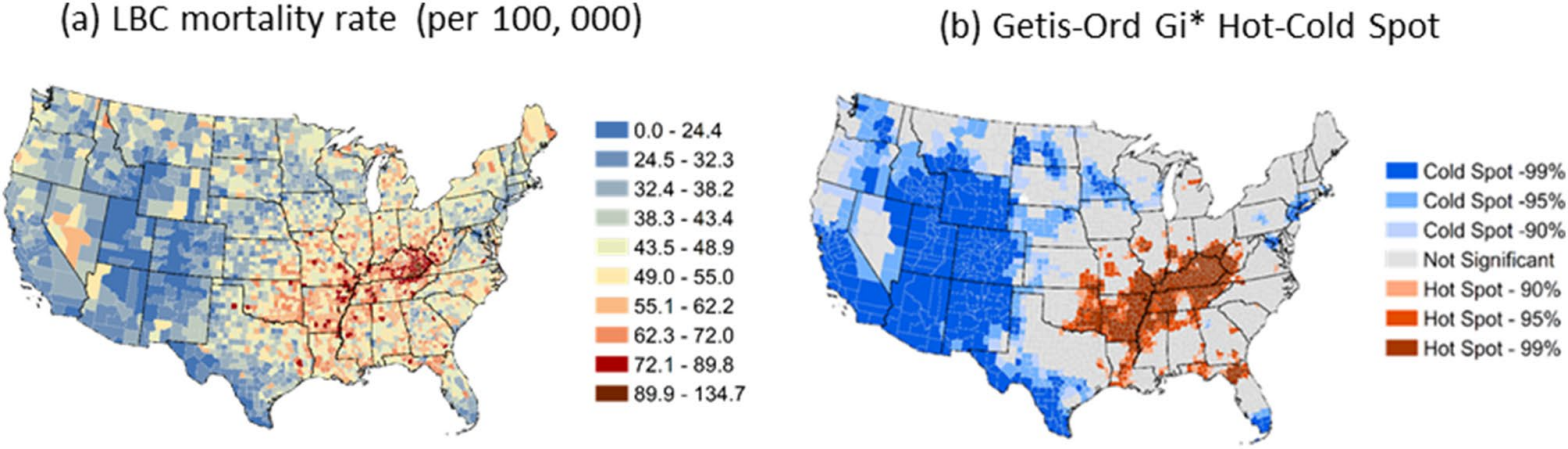
(**a**) County-level 5 years (2013–2017) average annual Lung and Bronchus Cancer (LBC) Mortality Rates, (**b**) The geographical clusters of counties with significant-high (hot spot—statistically significant positive z-scores, red color) or low (cold spot—statistically significant negative z-scores, sky blue colors) values of the Getis-Ord Gi* statistics for the LBC rate. LBC mortality rates and Getis-Ord Gi* Hot Spot maps were created in ArcGIS Desktop version 10.6.1^62^.

Before training, the data set (n = 3,107 counties) was randomly split using stratified random sampling^65^ into sub-sets of training (70%), validation (15%), and test data (15%). We used seven Gi-bins or clusters derived from Getis-Ord Gi* statistic of LBC mortality rates (Fig. 2a) as strata. The validation data was used to optimize the ML model parameters during the tuning and training processes. The test data set was used as the hold-out data to evaluate the model performance. The summary statistics and distribution of LBC mortality rate and risk factors in the training, validation, and test data sets are similar to those in the entire data set (Fig. S5a, b and Table S2).

##### Spatial regression models

The performance machine learning models were compared with three spatial regression models: spatial error, spatial Lag, and geographically weighted OLS (GW-OLS). A brief description of these models is given in supplementary information. For spatial regression analyses, "GWModel"^66^ and "spatialreg"^67^ packages in the R statistical computing environment were used^64^.

##### Machine learning base models

We trained the data with a generalized linear model (GLM), random forest (RF), Gradient boosting machine (GBM), extreme Gradient boosting machine (XGBoost), and Deep Neural Network (DNN) with several combinations of hyper-parameters. A brief description of all base learners is given in Supplementary information. During training, we used a Random Grid Search (RGS) to find the optimal parameter values for the base-learners to reduce over-fitting and enhance the prediction performance of the models^68^. The optimal hyperparameters were selected by conducting a grid-search using tenfold cross-validation (Supplementary Information Table S3). We used 0.001 and 2 for "stopping tolerance" and "stopping rounds" as early stopping parameters in the parameter tuning process. The best-performing model from each algorithm was selected according to their performance during tenfold cross-validation with different-parameters combinations. The root mean squared error (RMSE) was used as a performance matrix.

##### Stack-ensemble models

Ensemble machine learning with stack-generalization uses a higher-level model (meta–learner) to combine several lower-level models model as base-learners for better predict performance. Unlike the "bagging" in the random forest or "boosting" in Gradient boosting approaches that can only combine the same type of algorithm, stacked generalization can combine different algorithms to maximize the generalization accuracy. It uses the following three steps: (1) set up a list of base-learners (level-0 space) and a meta-learner (level-1 space), (2) train each of the base-learners and perform *K*-fold cross-validation predictions for each base-learner, and (3) use these predicted values to train the meta-learner and make new predictions. The base-level models often consist of different learning algorithms, and therefore stacking ensembles often combine heterogeneous algorithms. The *K*-fold cross-validation outputs of all base learners were then trained with two stacked ensemble models at the end. One ensemble contains all the sub-models of five learners (n = 147), and the other includes just the best-performing model from each learner. The GLM regression model was used as a meta-learner at level 1-space.

We used the "h2o" package^69^ in the R statistical computing environment^64^ to train, validate, and predict the GLM, RF, GBM, XGBoost, DNN, and stack-ensemble models.

##### Model performance

The performance of all base-learners and stack-ensemble models were evaluated with a hold-out-test data set. The diagnostic measures of prediction performance used here were the mean absolute error (MAE) (1), and the root mean square error (RMSE) (2). Also, we used observe versus predicted plots to visualize model performance and used simple linear regression between observed and predicted LBC-rates to judge model performance.

1 $$MAE = \frac{1}{n}\sum\limits_{i = 1}^{n} | (y_{i} - \hat{y})|$$

2 $$RMSE = \sqrt {\frac{1}{n}\sum\limits_{i = 1}^{n} [ y_{i} - \hat{y}]^{2}_{{^{{}} }} }$$

where *n* is the number of counties, and $y$ and $\hat{y}$ are observed and predicted LBC rates in county *i*.

We also calculated bias and variance of all spatial regression and ML models by resampling the training data set, repeating the model-building process, and deriving the average prediction error from the test data set. Bias represents how far away an average model prediction $\hat{f}\left( x \right)$ is far from the true $f\left( x \right)$, so, bias can be expressed as:

3 $$Bias \left( {\hat{f}\left( x \right)} \right) = E\left[ {\hat{f}\left( x \right)} \right] - f\left( x \right)$$

The variance represents how much a model prediction changes with different training data, i.e., variation in prediction due to random sampling:

4 $$Var \left( {\hat{f}\left( x \right)} \right) = E\left[ {\hat{f}\left( x \right)^{2} } \right] - E\left[ {\hat{f}\left( x \right)} \right]^{2}$$

So total expected error of a model prediction is composed of bias and variance:

5 $$Err\left( x \right) = Bias \left( {\hat{f}\left( x \right)} \right) + Var \left( {\hat{f}\left( x \right)} \right) + \sigma$$

#### Explainable AI

The Permutation Feature Importance (PFI) approach^70^ and Partial dependence plots (PDP)^71^ are primarily used to explain and visualize the output from simple machine learning models. Unlike traditional statistical methods, the output of the stacked ensemble model is difficult to interpret since it combines different ML algorithms^72^. Therefore, we created several agnostic "model explainers " to interpret the stack ensemble model's output at local and global scales. The "explainers" make a unified representation of a model for further analysis^37^.

##### Permutation-based feature importance

We adopted the "model agnostic" Permutation Feature Importance (PFI) approach^70^, which measures the increase in the prediction error (drop-out loss or RMSE) of the model after the feature values are permuted by breaking down the relationship between the feature and the true outcome. This probabilistic method automatically considers interaction effects for importance calculation^73^.

##### Partial dependence (PD), local-dependence (LD) and accumulated-local (AL) profiles

It is not easy to interpret complex machine learning algorithms by examining their coefficients. However, a partial dependence (PD) profile can interpret a machine learning model's output and visualize how the model's predictions are influenced by each predictor when all other predictors are being controlled. In these plots, the Y-axis value ($\widehat{y}$) is determined by the average of all possible model prediction values when the value of the objective predictor is at the value indicated on the X-axis.

Partial dependence plots can produce inaccurate interpretations if the predictors are strongly correlated^37^. As an alternative to partial dependence profiles, a new visualization approach, "accumulated local effects plots," has been proposed, which is unbiased and does not require this unreliable extrapolation with correlated predictors^74^. As accumulated-local (AL) profiles are related to local-dependence profiles (LD)^37^, both were applied to summarize the influence of an explanatory variable on the stack-ensemble model's predictions in this study.

##### Model agnostic greedy explanations of model predictions (breakDown)

We applied the break-down variable's contribution to visualize and describe how risk factors contribute to LBC mortality rates prediction locally (at the county level). The objective of this approach is to decompose model predictions into parts that can be attributed to particular variables^75^. The "Break-down Plots" proposed by Biecek and Burzykowski^37^ presents "variable attributions," *i.e*., "the decomposition of the model's prediction into contributions that can be attributed to different explanatory variables."

We used the "DALEX" package^76^ in R Statistical Computing Environment^64^ to create "explainers" for PFI, PD -, LD- and AL-profiles, and local variables' contribution in the best performing stack-ensemble model prediction.

## Results

### Exploratory data analysis

Figure 2a shows the spatial distribution of county-level, age-adjusted LBC rates, averaged over 5 years (2013–2017). There was a total of 146,193 LBC -related deaths in the US during this period. The South and Appalachian regions had mean LBC rates during the period 1998–2012 that were much higher than the national average of 65 death per 100,000. The highest mean mortality rates were observed in Union County in Florida, followed by several counties in the Appalachian region covering Kentucky, Tennessee, and West Virginia, respectively. Counties with the lowest LBC mortality rates were observed in Summit County, Utah.

The Getis-Ord Gi* hotspot analysis identifies statistically significant clusters of counties with a high mortality rate ("hot" clusters) in the South, mainly in the Mississippi basin and the southern Appalachian region (Fig. 2b). The "cold" clusters (or areas where the mortality rate was relatively low) occurred predominantly in the Midwest and the western part of the country. There were some other small cold clusters of counties in the northeastern coastal region.

The correlations between LBC mortality rate and risk factors are weak to moderate (Fig. S6). The correlations were positive for LBC mortality rate and smoking (*r* = 0.623, *p* < 0.001), PM_2.5_ (*r* = 0.425, *p* < 0.001), SO_2_ (*r* = 0.293, *p* < 0.001) and poverty (*r* = 0.394, *p* < 0.001), and negative for LBC mortality rate and percent Hispanic population (*r* =  − 0.364, *p* < 0.001) and median elevation (*r* =  − 0.443, *p* < 0.001). The mean LBC mortality was significantly lower in the large metro area than in other areas (Fig. S7a). For radon zones groups, mean LBC mortality rates were lower in radon zones-1 (Fig. S7b). For the last 10 years, counties producing coal showed significantly higher LBC mortality rates than other counties (Fig. S7c).

The bivariate global Moran's I show a positive association between LBC mortality rates and smoking, PM_25_, SO_2,_ and poverty activity and a negative association between the Hispanic population and median elevation (Fig. S8). The bivariate LMI cluster of LBC mortality rates and twelve risk factors are presented in maps in Fig. S9. The red color (High-High) in maps corresponds to significant clusters of high LBC mortality rates and high prevalence of risk factors. The light red color (High-Low) in maps resembles clusters of high LBC mortality rates and low prevalence of risk factors.

To see how the risk factors explained the spatial distribution LBC mortality rate in the conterminous USA, we calculated q-statistic (strength of SSH) of 15 risk factors which were sorted in the order: Smoking > SO_2_ > PM_25_ > Elevation > Ozone > Poverty > Hispanic population > NO_2_ > Population-65 yr > Black population > White population > Uninsured > Radon zone > Coal (yes/no) > Urban–Rural (Table 1). The q-value of smoking prevalence indicates a moderate stratified heterogeneity effect on LBC mortality rates distribution. Fourteen out of 15 variables exhibit low SSH.

**Table 1:** Association of each feature (risk factors) with LBC mortality rates (q-values).

Features	q-statistic	*p*-value
Smoking	0.3916	0.000
SO_2_	0.2240	0.000
PM_25_	0.2183	0.000
Elevation	0.2111	0.000
Ozone	0.2103	0.000
Poverty	0.1818	0.000
Hispanic population	0.1711	0.000
NO_2_	0.1524	0.000
Population > 65 yr	0.0889	0.000
Black population	0.0494	0.000
White population	0.0483	0.000
Uninsured	0.0424	0.000
Radon zone	0.0360	0.000
Coal (yes/no)	0.0223	0.000
Urban–Rural	0.0179	0.000

Q-statistics measures the strength of the stratified spatial heterogeneity (SSH).

### Base learners turning parameters

The optimum RF model had ntrees, max_depth, and sample_rate of 576, 30, and 06, respectively. The best GBM had ntrees = 500, col_sample_rate = 0.5, max_depth = 20, min_rows = 1.0. The best XGBoost model was found to have hyper-parameters of ntree = 350, max depth = 3, min_row = 50, col sample rate = 75%. The DNN model had three hidden layers. Each layer had 100 neurons with a "Tanh", activation function, with very low L1 regularization and L2 regularization values to add stability and reduce the risk of over-fitting. The optimum GLM model had alpha = 0 and lambda = 1.

### Performance of base learners and stack ensemble model

The MAE values varied from 6.06 to 7.00 per 100,000, which is lower than the minimum value of the observed LBC rate, 10.1 per 100,000. All five base-learners displayed only slight differences in their RMSE statistics. Among the base-learners, the RF and GBM models performed better than all the other learners during the training stage (Table 2). They had lower MAE and RMSE statistics and explained more than 95% of the variability in LBC mortality rates for the training data set. However, when the models were applied to the validation data set, they had relatively high MAE and RMSE statistics, indicating problems in generalizing their results beyond the training data set (i.e., generalization error).

**Table 2:** Mean absolute error (MAE), root mean squared error (RMSE) and the goodness of fit (R^2^) during the training, validation, and testing stages.

Models	Model types	Training	Validation	Test
		**MAE**		
Spatial lag model	Spatial regression	6.57	9.24	9.02
Spatial error model	Spatial regression	6.65	6.73	6.53
GW-OLS	Spatial regression	5.67	6.30	6.20
GLM	Base-learners	6.64	6.77	6.49
RF	Base-learners	1.90	6.45	6.16
GBM	Base-learners	1.21	6.54	6.20
XGBoost	Base-learners	2.21	6.53	6.41
DNN	Base-learners	6.01	7.47	7.00
The best of the family of the base learners	Stack-ensemble	2.23	6.43	6.08
All base learners	Stack-ensemble	3.95	6.39	6.06
		**RMSE**		
Spatial lag model	Spatial regression	8.69	12.17	11.46
Spatial error model	Spatial regression	8.82	9.12	8.35
GW-OLS	Spatial regression	7.56	8.65	8.09
GLM	Base-learners	8.80	9.17	8.31
RF	Base-learners	2.51	8.66	8.03
GBM	Base-learners	1.58	8.84	8.06
XGBoost	Base-learners	3.24	8.87	8.35
DNN	Base-learners	7.93	9.69	9.03
The best of the family of the base learners	Stack-ensemble	3.03	8.58	7.95
All base learners	Stack-ensemble	5.21	8.42	7.74
		**The goodness of fit (R**^**2**^**)**		
Spatial lag model	Spatial regression	0.59	0.33	0.33
Spatial error model	Spatial regression	0.58	0.54	0.55
GW-OLS	Spatial regression	0.69	0.59	0.58
GLM	Base-learners	0.58	0.54	0.56
RF	Base-learners	0.98	0.59	0.58
GBM	Base-learners	0.99	0.57	0.58
XGBoost	Base-learners	0.97	0.57	0.57
DNN	Base-learners	0.70	0.50	0.48
The best of the family of the base learners	Stack-ensemble	0.96	0.60	0.59
All base learners	Stack-ensemble	0.89	0.62	0.61

*GLM* generalized linear model, *RF* random forest, *GBM* graditent boosting machine, *XGBoost* eXtreme Gradient Boosting (XGBoost), *DNN* deep neural networks, *GW-OLS* geographically weighted OLS regression.

The performance of three spatial regression models, five base-learners and two stack-ensemble models, was further evaluated using a hold-out test data set (Table 2 and Fig. S9). The stack-ensemble model with all base learners (N = 147) improved prediction over the five base models (level-0 space) and three traditional spatial regression models. The improvement in the RMSE ranged between 2 and 32%. The R^2^ for the predicted versus the observed values for the test data set was 0.61 (Table 2). None of the base-learners successfully predicted the lowest and highest LBC rates for the hold-out test data, and they over-estimated low-values and under-estimated higher values (Fig. S10a–j).

When all models were rerun with ten randomly sampled trained data sets and validated with a test data set, we found the bias^2^ of RF, GBM, and the stack-estimable with all base-learners were significantly lower than other models (Fig. S11a). However, the prediction variance of these models with different training data sets was high (Fig. S11b). The highest bias^2^ and the lowest variance were obtained with the spatial lag, GLM, and spatial error models.

### Permutation-based variable importance

**Figure 3:**
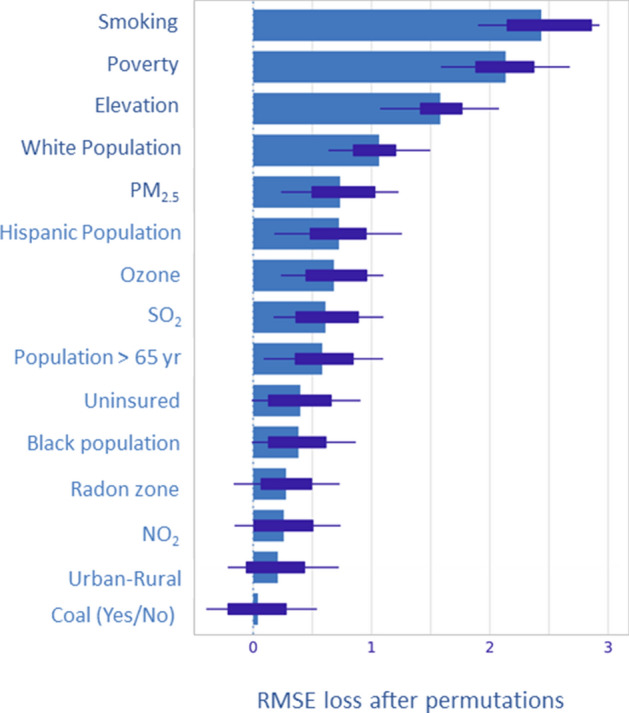
Permutation based feature ranking in stack-ensemble model.

The Feature Importance (the factor by which the RMSE is increased compared to the original model if a particular feature is permuted) of the best stack-ensemble model is shown in Fig. 3. Among the 15 risk factors, total smoking prevalence was identified as the most important variable, followed by poverty rate, elevation, percent white population, and PM_2.5_ in the contiguous US.

### Partial dependence (PD), local-dependence (LD), and accumulated-local (AL) profiles

**Figure 4:**
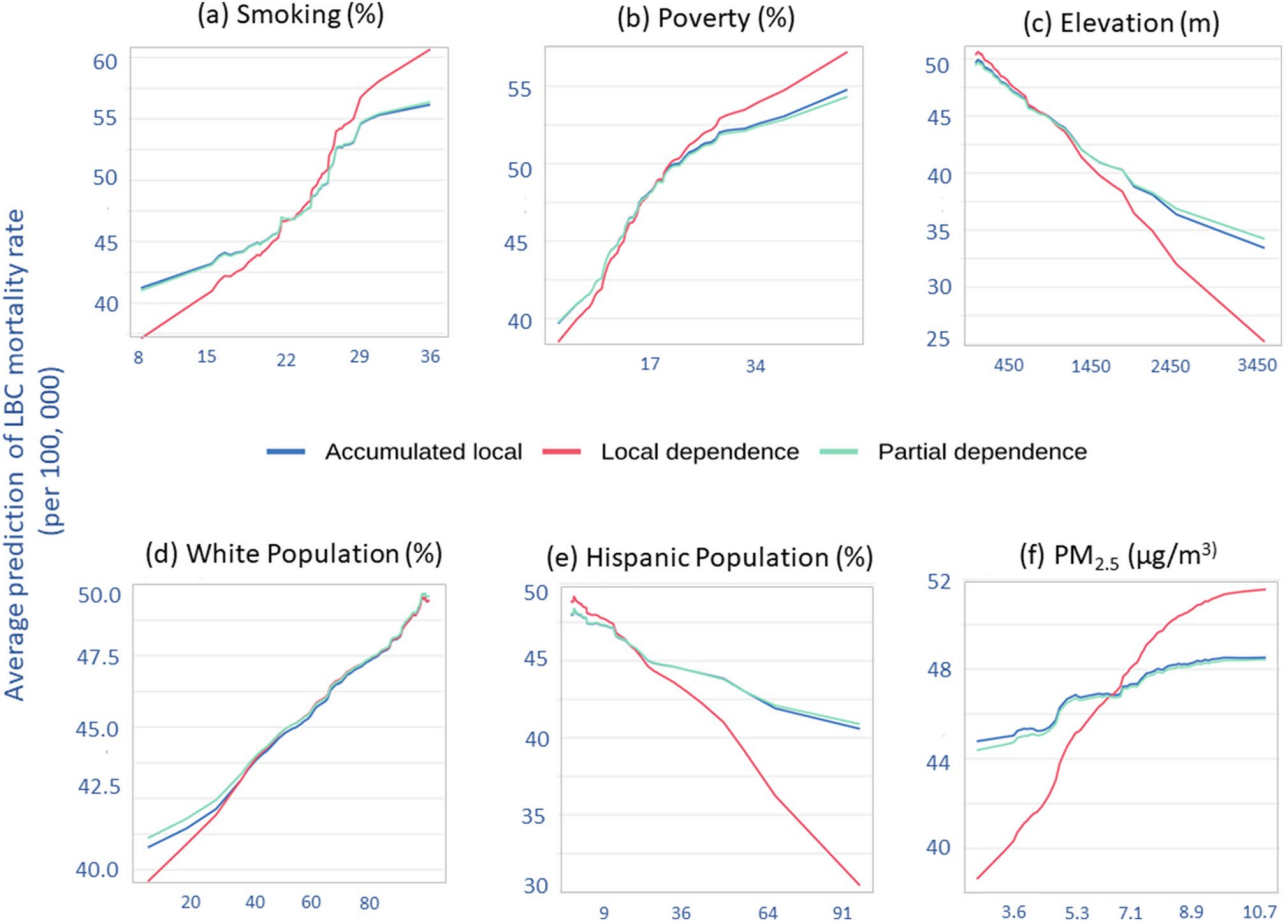
Partial-dependence, local-dependence, and accumulated-local profiles for the stack-ensemble model for the (**a**) smoking prevalence; (**b**) poverty rate; (**c**) elevation; (**d**) percentage white population; (**e**) percentage Hispanic population and (**f**) PM_2.5_.

Figure 4 shows partial-dependence, local-dependence, and accumulated-local profile plots of six important risk factors. Partial dependence plots help us understand the marginal effect of a feature (or subset thereof) on the predicted outcome. PD profiles offer a simple way to summarize a particular risk factor's effect on the LBC mortality rate. When other predictors were controlled for, the effects of smoking prevalence (Fig. 4a), poverty (Fig. 4b), percentage white population (Fig. 4d), and PM_2.5_ (Fig. 4f) showed a positive effect (blue lines) on predicted LBC mortality rates. However, elevation (Fig. 4c) and percentage Hispanic population (Fig. 4e) have a strong negative effect on expected LBC mortality rates.

**Figure 5:**
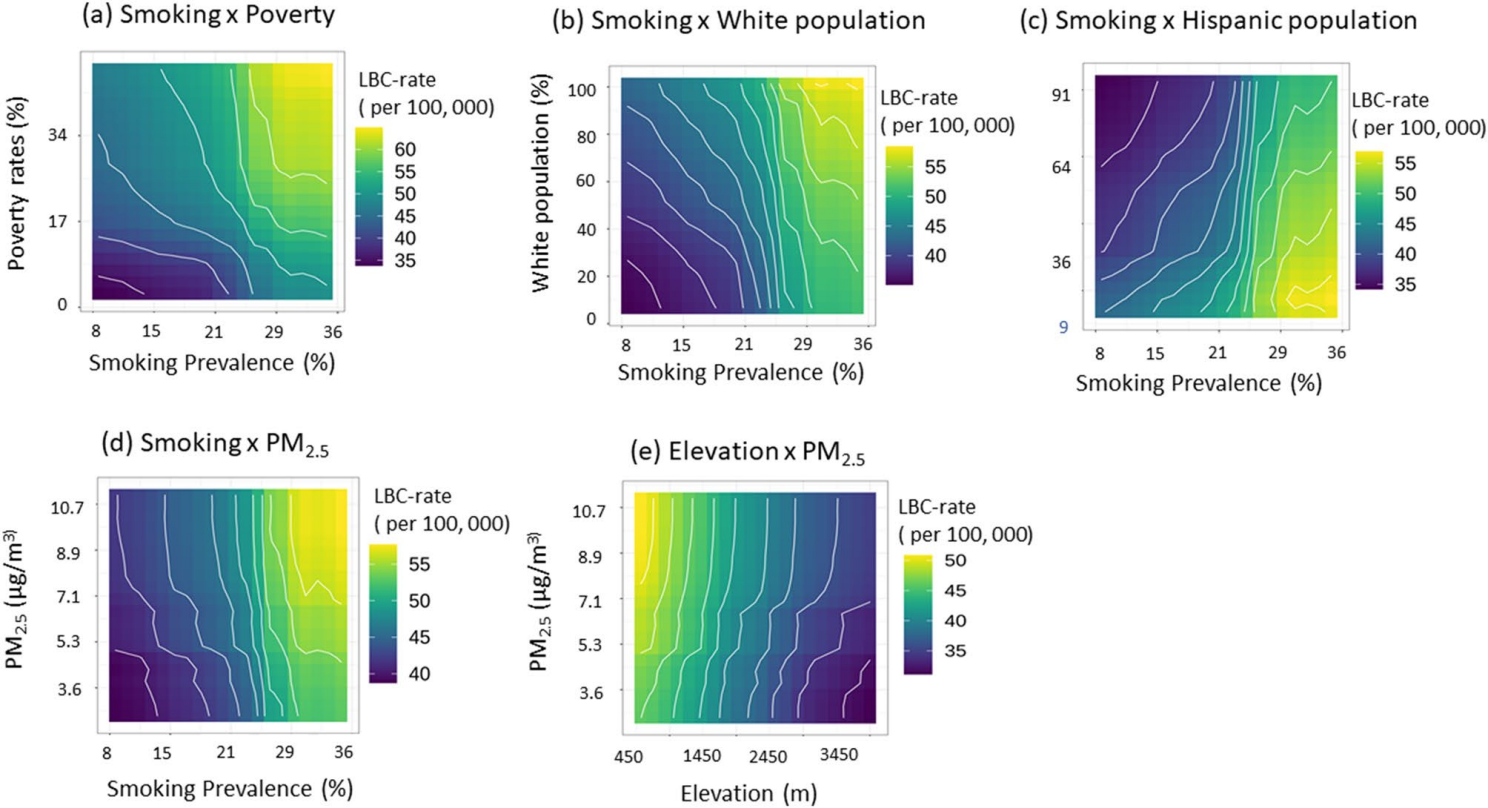
Two-variable partial dependence plots for the stack-ensemble modes for predicting LBC mortality rates. (**a**) Smoking versus poverty; (**b**) smoking versus white population; (**c**) smoking versus Hispanic population; (**d**) smoking vs. PM2.5; and (**e**) PM_2.5_ versus elevation.

Accumulated-local profiles are helpful in summarize an explanatory variable's influence on the model's predictions when explanatory variables are correlated. When the model is additive but, explanatory variables are correlated, neither PD nor LD profiles will adequately capture the explanatory variable's effect on the model's predictions^37^. However, the AL profile will provide a correct summary of the impact of variables on prediction. The AL and PD profiles (blue-lines Fig. 5) parallel each other for all six risk factors, suggesting that the stack-ensemble model is additive for these six explanatory variables.

The contour plot in Fig. 5 shows the dependence of the LBC mortality rate on the joint values of two risk factors when the effects of other risk factors are being controlled. When the average smoking prevalence is lower than ~ 30%, LBC rates are nearly independent of poverty, whereas, for smoking prevalence rates greater than ~ 30%, a strong dependence on poverty was observed (Fig. 5a). Similar positive interactions between smoking and the percent white population (Fig. 5b) and smoking and PM_2.5_ (Fig. 5d) were observed; since increases in these risk factors are associated with an increase in the LBC mortality rate. However, smoking prevalence and percent Hispanic population (Fig. 5c) and PM_2.5_ and Elevation (Fig. 5e) interacted in opposite ways in prediction.

### Break-down plots for additive attributions

**Figure 6:**
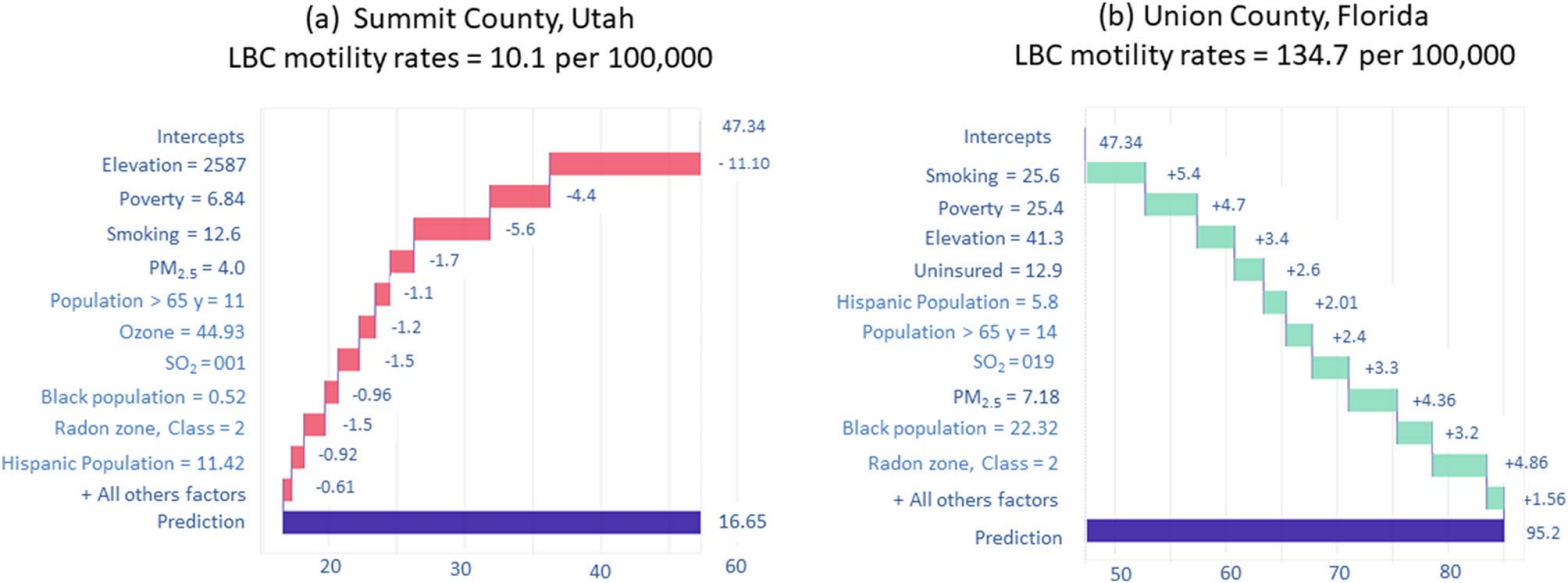
Break-down plots for the stack-ensemble model for the (**a**) Summit County, Utah, and Union County, Florida.

Break-down (BD) plots for a single observation are easy to understand, and several risk factors' contributions can be presented in a limited space. The BD. plots can be used to show "variable attributions," i.e., the decomposition of the model's prediction into contributions that can be attributed to different explanatory variables^37^. We selected two counties, Summit, Utah, and Union County, Florida, to explore the contribution of risk factors in two contrasting environments because the lowest and highest LBC mortality rates were observed in these counties. The median elevation in Summit and Union Counties are 2,587 and 47 m, respectively, and the prevalence of smoking and poverty in Summit County is lower than in Union county. The red and green bars in Fig. 6 indicate negative and positive changes in the mean predictions attributed to the risk factors. The most considerable negative contributions to predicting the LBC mortality rate for Summit County, Utah, come from elevation, smoking, and poverty (Fig. 6a). The contributions of the remaining other risk factors are smaller (in absolute values). For Union County, Florida, the predicted LBC mortality rate is attributed to the positive contribution of smoking, poverty, PM_2.5_, and radon-zone (Fig. 6b).

**Figure 7:**
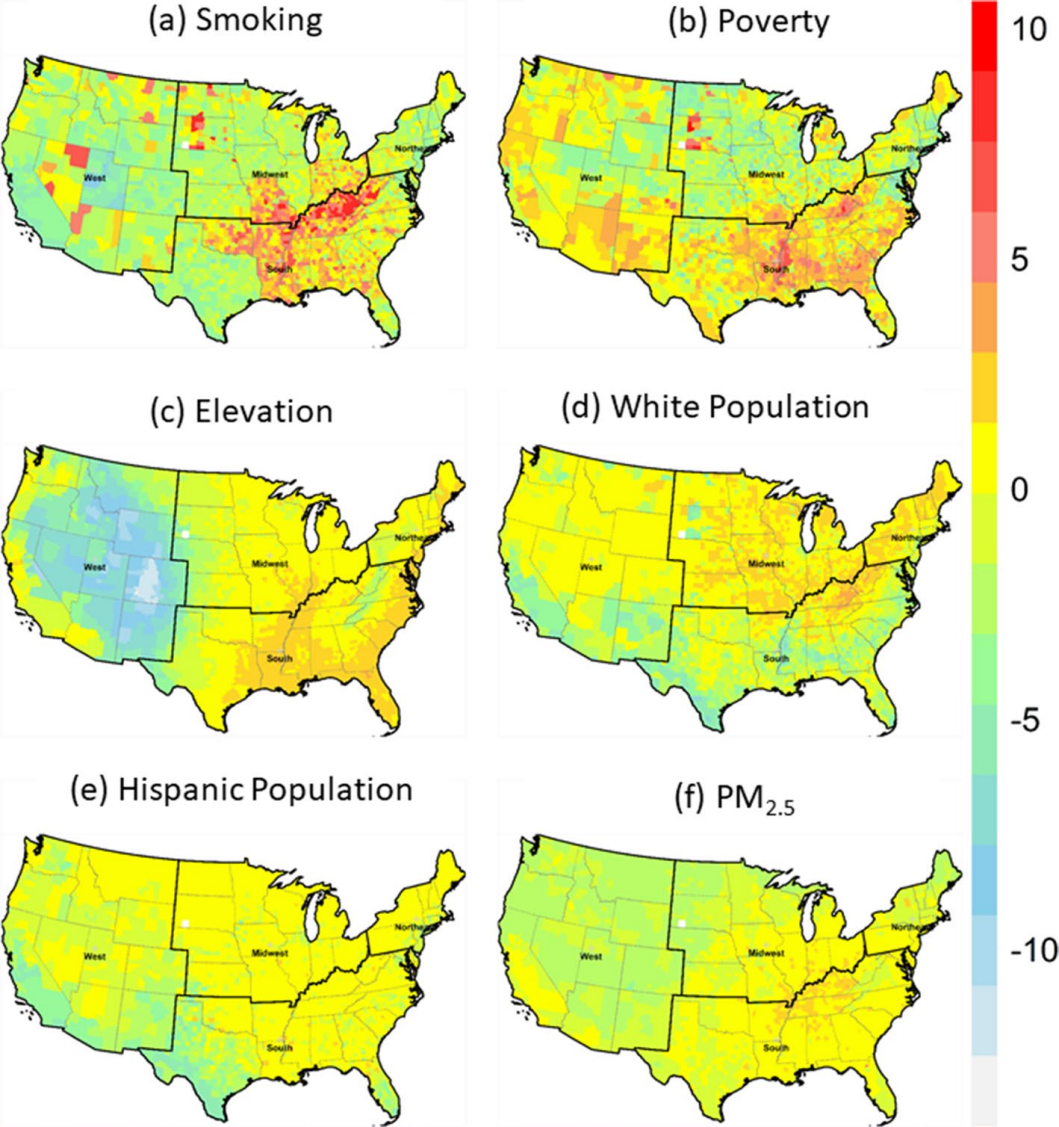
Spatial variation of local contribution of (**a**) smoking, (**b**) poverty (**c**) elevation, (**d**) white population, (**e**) Hispanic population, and (**f**) PM_2.5_ on the prediction of LBC mortality rates. The contribution of risk factors in each county's was calculated using "break-down plots" of stack-ensemble models. Maps were created in the R (version 4.1.1) Statistical Computing Environment^64^.

Figure 7 shows the spatial variability of the contribution of six risk factors for predicting LBC mortality rates in 3107 counties. A high positive contribution of smoking was observed in many counties in the Appalachians and the Mississippi Valley in the South and in the states of Missouri and Oklahoma (Fig. 7a). Poverty is identified as an important contributor in a large number of counties (Fig. 7b). The counties with high contributions from poverty on the LBC mortality rate are concentrated in the Appalachians and the Mississippi Valley in the South (Fig. 7b). Elevation, which is ranked the third most important risk factor overall, contributed negatively in many counties in the mountain area in the West, and Appalachian regions in the South, and the North East (Fig. 7c). In large numbers of counties in the Mid-West, North-East, and the Appalachian region in the South, percent white pollution showed a positive contribution to the predicted LBC mortality rate (Fig. 7d). A relatively higher Hispanic population negatively contributed to LBC mortality rate prediction in several counties in Texas, California, and New Mexico (Fig. 7e). Counties with a relatively low but positive contribution from PM_2.5_ are mostly located in the "Rust Belt**"** region in the Northeastern and Midwestern of the US and Appalachian areas in the South (Fig. 7f).

## Discussion

We demonstrated the potential use of stack-ensemble ML models and XAI to quantify and visualize the spatial variability of several risk factors' contributions to the LBC mortality rate across the conterminous USA. Geographically weighted (GW) models have widely been used to explore this relationship between risk factors and the LBC mortality rate^7,8,17–19^. However, GW models have limitations in exploring the spatial relationship since local regression coefficients are derived in locations (e.g., counties) based on the proximate area of interest and number of neighbors^77^. To overcome this limitation, XAI with local model-agnostic interpretability and break-down plots^37^ shows promise to explore risk factors' contribution to spatial variability LBC mortality rates.

In general, interpretable MI falls into two broad categories: personalized or prediction-level interpretation and dataset- or population-level interpretation, known as local and global interpretations, respectively^28^. The permutation-based feature importance, a global level-interpretation, identified smoking prevalence as the most important risk factor for LBC mortality. However, break-down plots of local model-agnostic showed a spatial variation in smoking's contributions to LBC mortality rate across the conterminous USA. In general, counties in the southern states, particularly in the Appalachian region and Mississippi Valley, have high smoking prevalence and LBC mortality rates^3,78–80^. The probability of smoking was strongly associated with compositional covariates: poverty, education, occupation, age, sex, race/ethnicity, nativity, employment, marital status, and household size^81^. Although cigarette smoking prevalence declined from 20.9% in 2005 to 14.0% in 2017, smoking is still a major cause of disease and death in the USA, accounting for more than 480,000 deaths every year, or about 1 in 5 deaths^4^. The high LBC mortality rates in the Appalachian region and Mississippi Valley can also be partly explained by high poverty rates, limited healthcare access, low educational attainment, and coal mining^82,83^. We identified county-level poverty rate as the second most important risk factor for LBC mortality across the contiguous US. Multivariate PD profile plots reveal a positive interaction between smoking and poverty rates since increasing both features leads to increased LBC mortality rates. The relationship between socioeconomic status and LBC mortality rates in the US is well established^13,82,84,85^. Access to health care is an economic issue, particularly in the US^7^. The socioeconomic status, such as poverty, determines early diagnosis and treatment and reduces the risk of death from LBC^86^. Percent population access to health insurance which is linked with poverty contributed strongly in predicting a high LBC mortality rate in Union County, Florida, which has the highest national LBC mortality rate.

Lung cancer incidence and mortality across the US were associated with the demographic composition^87^. In this study, we found that the percentage of the white or Hispanic population contributed positively and negatively, respectively, to the LBC mortality rate. Counties with a high proportion of white people in the Mid-West, North-East, and the Appalachian region in the South had higher LBC mortality rates than counties in the West with a relatively high proportion of Hispanics. Hispanics in the US have about a 50% lower incidence rate for lung cancer than the non-Hispanic white population^88^. Their presence contributed to lower LBC mortality rates in the western US generally^7^. The lower LBC incidence and mortality rates in this region are probably due to lower smoking rates in the Hispanic population^88^. We found a negative association between county-level smoking prevalence and the Hispanic population (*r* =  − 0.315, *p* < 0.001).

After smoking and poverty, median elevation ranked third in predicting LBC mortality nationally. In many mountainous counties in the West and North-East, elevation showed a negative contribution in prediction, which is consistent with the conceptual model of the impact of elevation on LBC mortality rates^89^ and the study of Kerry et al.^7^. Low atmospheric oxygen in higher elevation areas acts as an inhibitor of free radical damage and tumorigenesis, which may be responsible for low incidence respiratory cancers across the US's mountainous counties^89^.

The overall association between the LBC mortality rate and PM_2.5_ and SO_2_ was positive among the four air pollutants. The shared geographic area of high LBC mortality rate, smoking, poverty, and air pollution (PM_2.5_ and SO_2_) in the southeast and the Appalachian region indicate the association of these risk factors with higher LBC mortality rates. Other factors, such as poor diet, genetic susceptibility, and occupational exposures, may act independently or in concert with smoking or air pollution in determining LBC incidence and mortality^90^. Inferior air quality in these regions may synergistically contribute to a higher risk of lung cancer or respiratory illness^91,92^.

This study has some limitations. The county-level data inherent limitations since data are model-based estimates from the BRFSS telephone survey^93^. Furthermore, the LBC rate data used in this study contain errors due to the under-recording of lung cancer deaths, errors in population count, and covariates used in modeling. Besides the limitation of the data, the "post-doc explainable ML" model has some limitations^29^. The XAI is usually not suggested for high-stack discussion making due to its unreliable and unrealistic explanation of what the original model computes. However, it is recently being used in health sectors^36,94,95^. Very recently stack-ensemble model with model agnostic methods has been applied to identify factors influencing childhood blood lead levels^72^.

## Conclusions

To our knowledge, this study is the first one to apply XAI as "model greedy agnostic explanations of model predictions" or "break-down plot"^37^ in a stack-ensemble framework to explore the spatial variability of the contribution of several risk factors to LBC mortality rates. Application of XAI for understanding the spatial variability of the associations between LBC mortality rates and the risk factors may allow advanced research and policy development to understand underlying, spatially varying contributors to LBC mortality across US counties. This study shows strong potential for implementing XAI as a complement to or substitute for the traditional spatial regression models. This study's findings may lead to more tailored and effective prevention strategies from a policy perspective, which is critical, given the projected prevalence growth of LBC mortality rates in the coming decades.

## Supplementary Information

Supplementary Information 1.

## Data Availability

The data sets generated during this study are available from the corresponding author upon reasonable request.

## References

[1] Bray F (2018). Global cancer statistics 2018: GLOBOCAN estimates of incidence and mortality worldwide for 36 cancers in 185 countries. CA: Cancer J. Clin..

[2] Wang H (2016). Global, regional, and national life expectancy, all-cause mortality, and cause-specific mortality for 249 causes of death, 1980–2015: A systematic analysis for the global burden of disease study 2015. Lancet.

[3] Siegel RL, Miller KD, Jemal A (2019). Cancer statistics, 2019. CA: Cancer J. Clin..

[4] Centers for Disease Control and Prevention (CDC). * U.S. Cancer Statistics Working Group.* https://www.cdc.gov/cancer/lung/statistics/ (2019).

[5] Mokdad AH (2017). Trends and patterns of disparities in cancer mortality among US counties, 1980–2014. JAMA.

[6] Centers for Disease Control and Prevention (CDC) (2011). State-specific trends in lung cancer incidence and smoking—United States, 1999–2008. MMWR Morb. Mortal. Wkly Rep..

[7] Kerry R, Goovaerts P, Ingram B, Tereault C (2019). Spatial analysis of lung cancer mortality in the American west to improve allocation of medical resources. Appl. Spat. Anal. Policy.

[8] Moore JX, Akinyemiju T, Wang HE (2017). Pollution and regional variations of lung cancer mortality in the United States. Cancer Epidemiol..

[9] Albano JD (2007). Cancer mortality in the United States by Education level and race. JNCI: J. Natl. Cancer Inst..

[10] Winkler V, Ng N, Tesfaye F, Becher H (2011). Predicting lung cancer deaths from smoking prevalence data. Lung Cancer.

[11] Jeon J (2018). Smoking and lung cancer mortality in the United States from 2015 to 2065: A comparative modeling approachsmoking and lung cancer mortality in the United States From 2015 to 2065. Ann. Intern. Med..

[12] Singh GK, Siahpush M, Williams SD (2012). Changing urbanization patterns in US lung cancer mortality, 1950–2007. J. Commun. Health.

[13] Singh GK, Miller BA, Hankey BF (2002). Changing area socioeconomic patterns in US cancer mortality, 1950–1998: Part II—Lung and colorectal cancers. J. Natl. Cancer Inst..

[14] Quick H (2019). Estimating county-level mortality rates using highly censored data from CDC WONDER. Prev. Chronic Dis..

[15] Tiwari RC (2004). A new method of predicting US and state-level cancer mortality counts for the current calendar year. CA: Cancer J. Clin..

[16] Wingo P, Landis S, Parker S, Bolden S, Heath C (1998). Using cancer registry and vital statistics data to estimate the number of new cancer cases and deaths in the United States for the upcoming year. J. Reg. Manag..

[17] Hu L, Griffith D, Chun Y (2018). Space-time statistical insights about geographic variation in lung cancer incidence rates: Florida, USA, 2000–2011. Int. J. Environ. Res. Public Health.

[18] Hu Z, Baker E (2012). Geographical analysis of lung cancer mortality rate and PM2.5 using global annual average PM2.5 grids from MODIS and MISR aerosol optical depth. J. Geosci. Environ. Prot..

[19] Hystad P (2012). Spatiotemporal air pollution exposure assessment for a Canadian population-based lung cancer case-control study. Environ. Health.

[20] Rajpurkar, P. *et al.* Chexnet: Radiologist-level pneumonia detection on chest x-rays with deep learning. arXiv preprint http://arxiv.org/abs/1711.05225 (2017).

[21] Christensen, T., Frandsen, A., Glazier, S., Humpherys, J. & Kartchner, D. Machine Learning Methods for Disease Prediction with Claims Data. In *2018 IEEE International Conference on Healthcare Informatics (ICHI)* 467–4674. 10.1109/ICHI.2018.00108 (2018).

[22] Hsieh MH (2018). Comparison of machine learning models for the prediction of mortality of patients with unplanned extubation in intensive care units. Sci. Rep..

[23] Weng SF, Vaz L, Qureshi N, Kai J (2019). Prediction of premature all-cause mortality: A prospective general population cohort study comparing machine-learning and standard epidemiological approaches. PLoS ONE.

[24] Agrawal A, Misra S, Narayanan R, Polepeddi L, Choudhary A (2012). Lung cancer survival prediction using ensemble data mining on seer data. Sci. Program..

[25] Zhai B, Chen J (2018). Development of a stacked ensemble model for forecasting and analyzing daily average PM2.5 concentrations in Beijing, China. Sci. Total Environ..

[26] Wang Z, Wang K, Liu Z, Wang X, Pan S (2018). A cognitive vision method for insect pest image segmentation. IFAC-PapersOnLine.

[27] Ma Z, Wang P, Gao Z, Wang R, Khalighi K (2018). Ensemble of machine learning algorithms using the stacked generalization approach to estimate the warfarin dose. PLoS ONE.

[28] Murdoch WJ, Singh C, Kumbier K, Abbasi-Asl R, Yu B (2019). Definitions, methods, and applications in interpretable machine learning. Proc. Natl. Acad. Sci..

[29] Rudin C (2019). Stop explaining black box machine learning models for high stakes decisions and use interpretable models instead. Nat. Mach. Intell..

[30] Stiglic G (2020). Interpretability of machine learning-based prediction models in healthcare. WIREs Data Min. Knowl. Discov..

[31] Hall P, Gill N (2019). An Introduction to Machine Learning Interpretability.

[32] Gunning D, Aha D (2019). DARPA’s explainable artificial intelligence (XAI) program. AI Mag..

[33] Strumbelj E, Kononenko I (2010). An efficient explanation of individual classifications using game theory. J. Mach. Learn. Res..

[34] Ribeiro, M. T., Singh, S. & Guestrin, C. Model-agnostic interpretability of machine learning. *arXiv preprint* arxiv:1606.05386 (2016).

[35] Kumarakulasinghe, N. B., Blomberg, T., Liu, J., Leao, A. S. & Papapetrou, P. Evaluating Local Interpretable Model-Agnostic Explanations on Clinical Machine Learning Classification Models. In *2020 IEEE 33rd International Symposium on Computer-Based Medical Systems (CBMS)* 7–12. 10.1109/CBMS49503.2020.00009 (2020).

[36] De Sousa IP, Vellasco MMBR, Da Silva EC (2019). Local interpretable model-agnostic explanations for classification of lymph node metastases. Sensors.

[37] Biecek P, Burzykowski T (2021). Explanatory Model Analysis: Explore, Explain, and Examine Predictive Models.

[38] NCHS (2014). National Vital Statistics System: Multiple Cause of Death Data File, 1980–2014.

[39] Wingo PA (2003). Long-term trends in cancer mortality in the United States, 1930–1998. Cancer: Interdiscip. Int. J. Am. Cancer Soc..

[40] Murphy, S., Xu, J. & Kochanek, K. Deaths: Final data for 2010. National vital statistics reports. *National Center for Health Statistics* 61 (2013). 24979972

[41] Stekhoven, D. J. Using the missForest package. *R package*, 1–11 (2011).

[42] Institute for Health Metrics and Evaluation (IHME). *United States Smoking Prevalence by County 1996–2012. Seattle, United States of America: Institute for Health Metrics and Evaluation (IHME).* http://ghdx.healthdata.org/record/ihme-data/united-states-smoking-prevalence-county-1996-2012 (2014).

[43] Dwyer-Lindgren L (2016). US county-level trends in mortality rates for major causes of death, 1980–2014 US county-level trends in mortality rates for major causes of death US county-level trends in mortality rates for major causes of death. JAMA.

[44] Robert Wood Johnson Foundation. Health Ranking *2020 Measures.* University of Wisconsin Population Health Institute. https://www.countyhealthrankings.org/explore-health-rankings/measures-data-sources/2020-measures (2020).

[45] United States Census. *Small Area Income and Poverty Estimates (SAIPE) Program.* https://www.census.gov/programs-surveys/saipe/data/datasets.html (2018).

[46] Bell WR, Basel WW, Maples JJ (2016). An overview of the US census Bureau’s small area income and poverty estimates program. Anal. Poverty Data Small Area Estim..

[47] Robert Wood Johnson Foundation. *The County Health Rankings. * University of Wisconsin Population Health Institute*.* https://www.countyhealthrankings.org/explore-health-rankings/rankings-data-documentation (2020).

[48] Bowers, L., Gann, C. & Upton, R. Small area health insurance estimates: 2016. *Small Area Estimates. Current Population Reports* (accessed 31 July 2018); https://www.census.gov/programs-surveys/sahie.html (2018).

[49] United States Census. * Intercensal County Estimates by Age, Sex, Race: 1980–1989.* https://www.census.gov/data/datasets/time-series/demo/popest/1980s-county.html (2015).

[50] Centers for Disease Control and Prevention (CDC). *Daily PM2.5 Concentrations All County, 2001–2016.* https://data.cdc.gov/Environmental-Health-Toxicology/Daily-PM2-5-Concentrations-All-County-2001-2016/7vdq-ztk9 (2020).

[51] Berrocal VJ, Gelfand AE, Holland DM (2012). Space-time data fusion under error in computer model output: An application to modeling air quality. Biometrics.

[52] Geddes JA, Martin RV, Boys BL, Donkelaar AV (2016). Long-term trends worldwide in ambient NO_2_ concentrations inferred from satellite observations. Environ. Health Perspect..

[53] Fioletov V (2017). Multi-source SO_2_ emission retrievals and consistency of satellite and surface measurements with reported emissions. Atmos. Chem. Phys..

[54] Centers for Disease Control and Prevention (CDC). *Daily County-Level Ozone Concentrations, 2001–2016.* https://data.cdc.gov/Environmental-Health-Toxicology/Daily-County-Level-Ozone-Concentrations-2001-2016/kmf5-t9yc (2020).

[55] U.S. Environmental Protection Agency (USEPA). *EPA Map of Radon Zones Including State Radon Information and Contacts.* https://19january2017snapshot.epa.gov/radon/find-information-about-local-radon-zones-and-state-contact-information_html#radonmap (2020).

[56] U.S. Department of Agriculture (USDA). *Rural-Urban Continuum Codes.* https://www.ers.usda.gov/data-products/rural-urban-continuum-codes.aspx (2013).

[57] Ingram, D. D. & Franco, S. J. *2013 NCHS Urban-Rural Classification Scheme for Counties. **Vital Health Stat. 2.* 1–73 (2014). 24776070

[58] U.S. Energy Information Administration (EIA). *Coal Data Browser.* https://www.eia.gov/coal/data/browser/ (2018).

[59] U.S. Geological Survey (USGS). USGS EROS archive—Digital elevation—Shuttle radar topography mission (SRTM) void filled 1 arc-second global. *Earth Resour. Obs. Sci. Cent.* (2018).

[60] Hardy OJ, Vekemans X (1999). Isolation by distance in a continuous population: Reconciliation between spatial autocorrelation analysis and population genetics models. Heredity.

[61] Cliff AD, Ord K (1970). Spatial autocorrelation: A review of existing and new measures with applications. Econ. Geogr..

[62] Environmental Systems Research Institute (ESRI). *ArcGIS Desktop: Release 10.6.1* (2019).

[63] Wang J-F, Zhang T-L, Fu B-J (2016). A measure of spatial stratified heterogeneity. Ecol. Ind..

[64] R Core Team. *R: A Language and Environment for Statistical Computing*. R Foundation for Statistical Computing. https://www.R-project.org/ (2021).

[65] de Vries P.G. (1986). Stratified Random Sampling. In: Sampling Theory for Forest Inventory. Sampling Theory for Forest Inventory.

[66] Gollini, I., Lu, B., Charlton, M., Brunsdon, C. & Harris, P. GW model: An R package for exploring spatial heterogeneity using geographically weighted models. *arXiv preprint* arxiv:1306.0413 (2013).

[67] Bivand, R. & Piras, G. spatialreg: Spatial regression analysis. R package version, 1.1–5 (2019).

[68] Hamidieh K (2018). A data-driven statistical model for predicting the critical temperature of a superconductor. Comput. Mater. Sci..

[69] Aiello, S., Kraljevic, T., Maj, P. & Team, C. F. T. H. O. A. H_2_O: R Interface for H_2_O. R Package Version, Vol. 3 (2016).

[70] Fisher, A., Rudin, C. & Dominici, F. Model class reliance: Variable importance measures for any machine learning model class, from the “Rashomon” perspective. *arXiv preprint* arxiv:1801.01489 (2018).

[71] Friedman JH (2001). Greedy function approximation: A gradient boosting machine. Ann. Stat..

[72] Liu X, Taylor MP, Aelion CM, Dong C (2021). Novel application of machine learning algorithms and model-agnostic methods to identify factors influencing childhood blood lead levels. Environ. Sci. Technol..

[73] Molnar, C. *Interpretable Machine Learning*. Lulu.com (2020).

[74] Apley DW, Zhu J (2020). Visualizing the effects of predictor variables in black box supervised learning models. J. Roy. Stat. Soc.: Ser. B (Stat. Methodol.).

[75] Staniak, M. & Biecek, P. Explanations of model predictions with live and breakDown packages. *arXiv preprint* arxiv:1804.01955 (2018).

[76] Biecek P (2018). DALEX: Explainers for complex predictive models in R. J. Mach. Learn. Res..

[77] Hipp JA, Chalise N (2015). Spatial analysis and correlates of county-level diabetes prevalence, 2009–2010. Prev. Chronic Dis..

[78] Lengerich EJ (2005). Cancer incidence in Kentucky, Pennsylvania, and West Virginia: Disparities in Appalachia. J. Rural Health.

[79] Wingo PA (2008). Cancer in Appalachia, 2001–2003. Cancer.

[80] Dwyer-Lindgren L (2014). Cigarette smoking prevalence in US counties: 1996–2012. Popul. Health Metr..

[81] Chahine T, Subramanian SV, Levy JI (2011). Sociodemographic and geographic variability in smoking in the U.S.: A multilevel analysis of the 2006–2007 current population survey, tobacco use supplement. Soc. Sci. Med..

[82] Mejia de Grubb, M. C. *et al.**Socioeconomic, Environmental, and Geographic Factors and US Lung Cancer Mortality, 1999–2009*. 10.15212/FMCH.2017.0108 (2017).

[83] Appalachian Regional Commission and West Virginia University. Office for Social Environment and Health Research. Underlying Socioeconomic Factors Influencing Health Disparities in the Appalachian Region: Final Report. Mary Babb Randolph Cancer Center/Office for Social Environment and Health Research, Dept. of Community Medicine, Robert C. Byrd Health Sciences Center, West Virginia University. http://purl.access.gpo.gov/GPO/LPS100135 (2008).

[84] Boscoe FP (2014). The relationship between area poverty rate and site-specific cancer incidence in the United States. Cancer.

[85] Boscoe FP, Henry KA, Sherman RL, Johnson CJ (2016). The relationship between cancer incidence, stage and poverty in the United States. Int. J. Cancer.

[86] Woods L, Rachet B, Coleman M (2006). Origins of socio-economic inequalities in cancer survival: A review. Ann. Oncol..

[87] Tabatabai MA (2016). Racial and gender disparities in incidence of lung and bronchus cancer in the United States: A longitudinal analysis. PLoS ONE.

[88] Haile RW (2012). A review of cancer in US Hispanic populations. Cancer Prev. Res..

[89] Simeonov KP, Himmelstein DS (2015). Lung cancer incidence decreases with elevation: Evidence for oxygen as an inhaled carcinogen. PeerJ.

[90] Malhotra J, Malvezzi M, Negri E, La Vecchia C, Boffetta P (2016). Risk factors for lung cancer worldwide. Eur. Respir. J..

[91] Hamra GB (2014). Outdoor particulate matter exposure and lung cancer: A systematic review and meta-analysis. Environ. Health Perspect..

[92] Huang F, Pan B, Wu J, Chen E, Chen L (2017). Relationship between exposure to PM2.5 and lung cancer incidence and mortality: A meta-analysis. Oncotarget.

[93] Barker LE, Kirtland KA, Gregg EW, Geiss LS, Thompson TJ (2011). Geographic distribution of diagnosed diabetes in the US: A diabetes belt. Am. J. Prev. Med..

[94] Song X (2020). Cross-site transportability of an explainable artificial intelligence model for acute kidney injury prediction. Nat. Commun..

[95] Lauritsen SM (2020). Explainable artificial intelligence model to predict acute critical illness from electronic health records. Nat. Commun..

