# Supplementary material for: Explainable artificial intelligence (XAI) for exploring spatial variability of lung and bronchus cancer (LBC) mortality rates in the contiguous USA
Source: Sci Rep. 2021 Dec 16;11:24090. doi: 10.1038/s41598-021-03198-8 (PMC8677843; doi:10.1038/s41598-021-03198-8)
Supplement: Supplementary file 1 — Supplementary Information 1. [file 41598_2021_3198_MOESM1_ESM.pdf]

Supplementary information

Explainable Artificial Intelligence (XAI) for Exploring Spatial Variability of Lung and Bronchus Cancer (LBC) Mortality Rates in the contiguous USA

Zia U. Ahmed*, Kang Sun, Michael Shelly, and Lina Mu

Zia U. Ahmed*
Research and Education in Energy, Environment and Water (RENEW) Institute
108 Cooke Hall, University at Buffalo
Buffalo, NY 14260
Phone: 716-645-1405
Email: zahmed2@buffalo.edu

Kang Sun
Research and Education in Energy, Environment and Water (RENEW) Institute
106 Cooke Hall, University at Buffalo
Buffalo, NY 14260
Phone: 716-645-6167
Email: kangsun@buffalo.edu

Michael Shelly
Research and Education in Energy, Environment and Water (RENEW) Institute
108 Cooke Hall, University at Buffalo
Buffalo, NY 14260
Phone: 716-645-5900
Email: mshelly@buffalo.edu

Lina Mu
Department of Epidemiology and Environmental Health
273A Farber Hall, University at Buffalo
Buffalo, NY 14214
Phone: 716-829-5381

.

Supplementary information

The Supplementary information comprises 27 pages and includes the following supplementary figures and tables:

List of Figures:

Figure S1. Long-term average county-level (a) smoking prevalence; (b) poverty, and (c) uninsured percentage

Figure S2. Maps showing the 5 years mean of annual data in a county for percent (a) white, (b) black, (c) Hispanic/Latino, and (d) population aged 65 and older.

Figure S3. Long-term annual mean of county-level (a) Fine Particulate Matter ($PM_{2.5}$); (b) nitrogen dioxide (NO_2); (c) Sulfur dioxide (SO_2) and (d) ozone concentrations

Figure S4. (a) Randon zones; (b) Urban-rural counties (c) Coal production counties (SO_2) and (d) median elevation

Figure S5. (a) spatial distribution, training, validation, and test counties, and (b) distribution of LBC mortality rate in all, training, validation, and test data.

Figure S6. Pearson correlation matrix plots. In this plot, correlation coefficients are colored according to the value. The correlation matrix can be also reordered according to the degree of association between variables. Positive correlations are displayed in blue and negative correlations in red color. The color intensity and the size of the circle are proportional to the correlation coefficients. On the right side of the correlogram, the legend color shows the correlation coefficients and the corresponding colors. The correlations with p-value > 0.05 are considered as insignificant. In this case, the correlation coefficient values are left blank.

Figure S7. Mean LBC mortality rates in relation to the (a) urban-rural area; (b) radon-sones and (c) coal productions.

Figure S8. Global bivariate Moran's I

Figure S9. Bivariate LMI cluster of LBC mortality rates and twelve risk factors.

Figure S10. 1:1 plot of observed versus predicted LBC mortality rate in 667 test counties. All models were trained and validated with data from 2171 and 468 counties, respectively.

Figure S11. $Bias^2$ and variance of spatial regression and machine learning models

List of Tables

Table S1. Data source and years LBC mortality rates and risk-factors

Table S2. Summary statistics of mean LBC mortality rates and risk factors of all, training, validation and test counties.

Table S3. List of hyper-parameters used to conduct the grid-search and to select the best parameters for the generalized linear model (GLM), distributed random forest (DRF), extreme gradient boosting machine (XGBoost), and Deep Neural Network (DNN).

Table S4. Summary results of ordinary least square (OLS), spatial lag , spatial error geographically weighted ordinary least squares regression (GW-OLS) models.

List of Texts

Text 1: Hot Spot Analysis (Getis-Ord Gi*)

Text 2: Bivariate Moran-I

Text 3: Spatial stratified heterogeneity (SSH**)**

Text 4: Spatial Lag Models

Text 5: Spatial Error Models

Text 6: Geographically Weighted OLS Regression (GW-OLS).

Text 7: Base Machine Learning Models

Text 7.1. *Generalized Linear Model (GLM*)

Text 7.2: *Random Forest (RF)*

Text 7.3: *Gradient boosting machine (GBM)*

Text 7.4: *Extreme Gradient boosting (XGBoost)*

Text 7.5. *Deep Neural Network (DNN)*

Text 8: Stack-Ensemble Model

Figure S1. Long-term average county-level (a) smoking prevalence; (b) poverty, and (c) uninsured percentage. Maps were created in the R (version 4.1.1) Statistical Computing Environment [1].

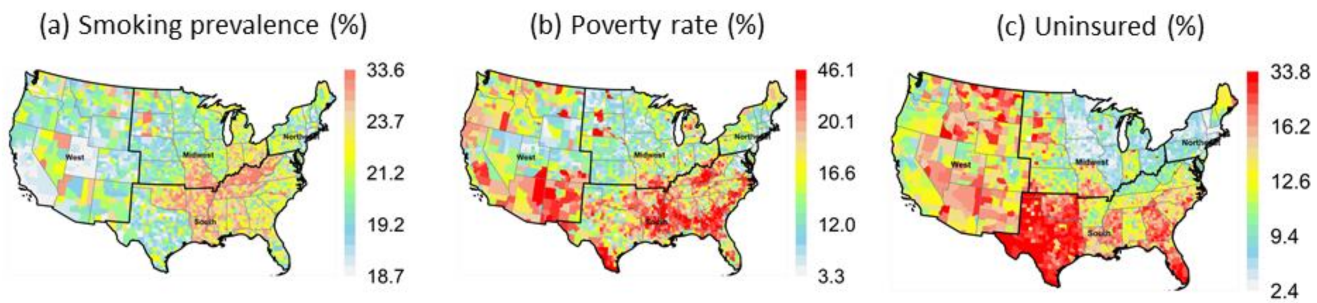

Figure S2. Maps showing the 5 year mean of annual data in a county for percent (a) white, (b) black, (c) Hispanic/Latino, and (d) population aged 65 and older. Maps were created in the R (version 4.1.1) Statistical Computing Environment [1].

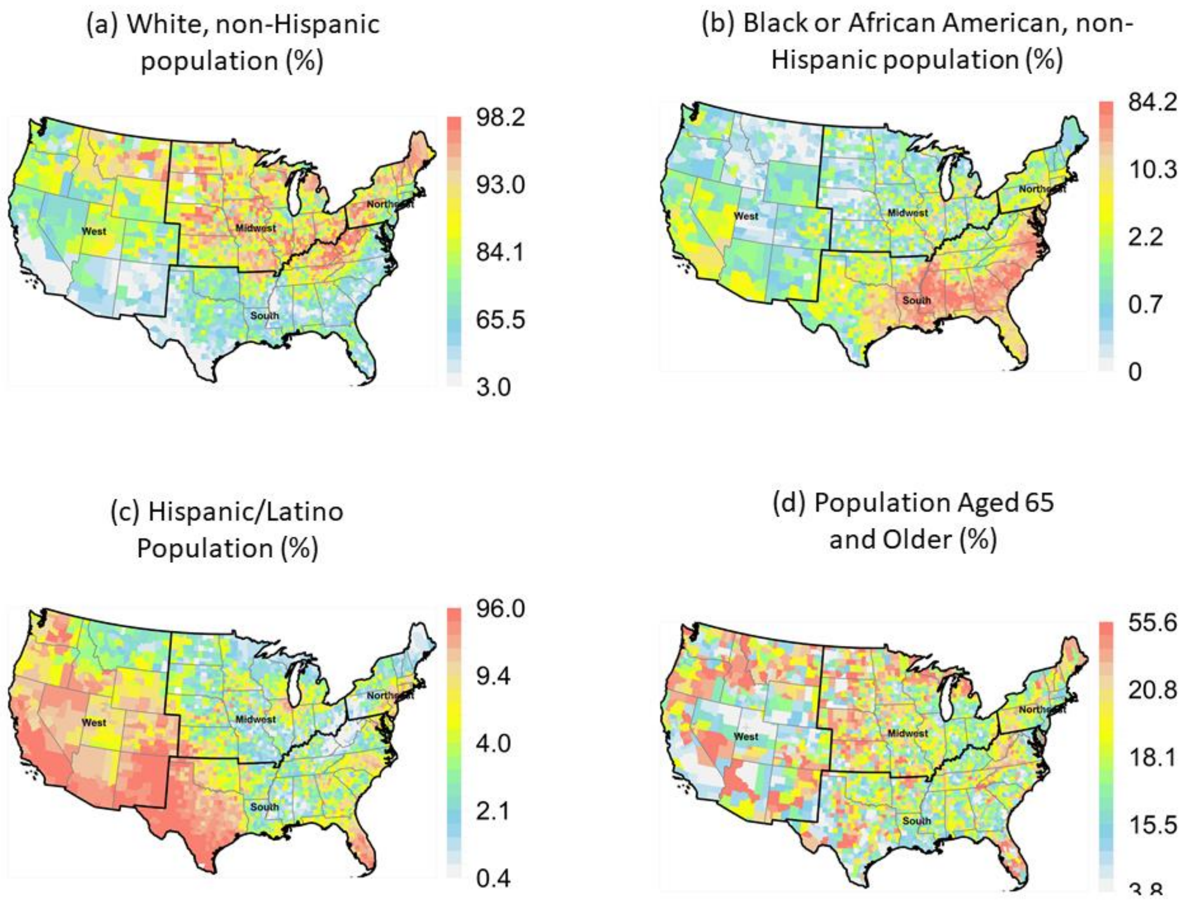

Figure S3. Long-term annual mean of county-level (a) Fine Particulate Matter ($PM_{2.5}$); (b) nitrogen dioxide (NO_2); (c) Sulfur dioxide (SO_2) and (d) ozone concentrations**.** Maps were created in the R (version 4.1.1) Statistical Computing Environment [1].

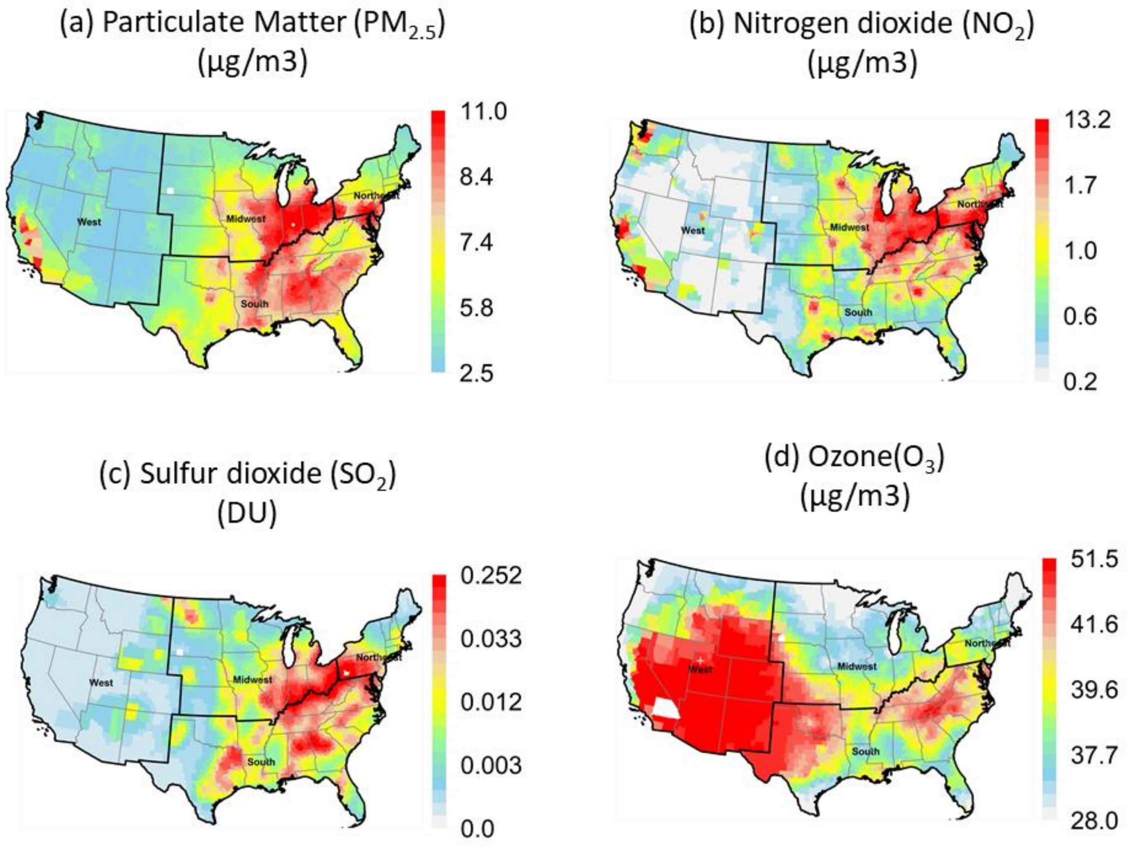

Figure S4. (a) Randon zones; (b) Urban-rural counties (c) Coal producing counties (SO_2) and (d) median elevation**.** Maps were created in the R (version 4.1.1) Statistical Computing Environment [1].

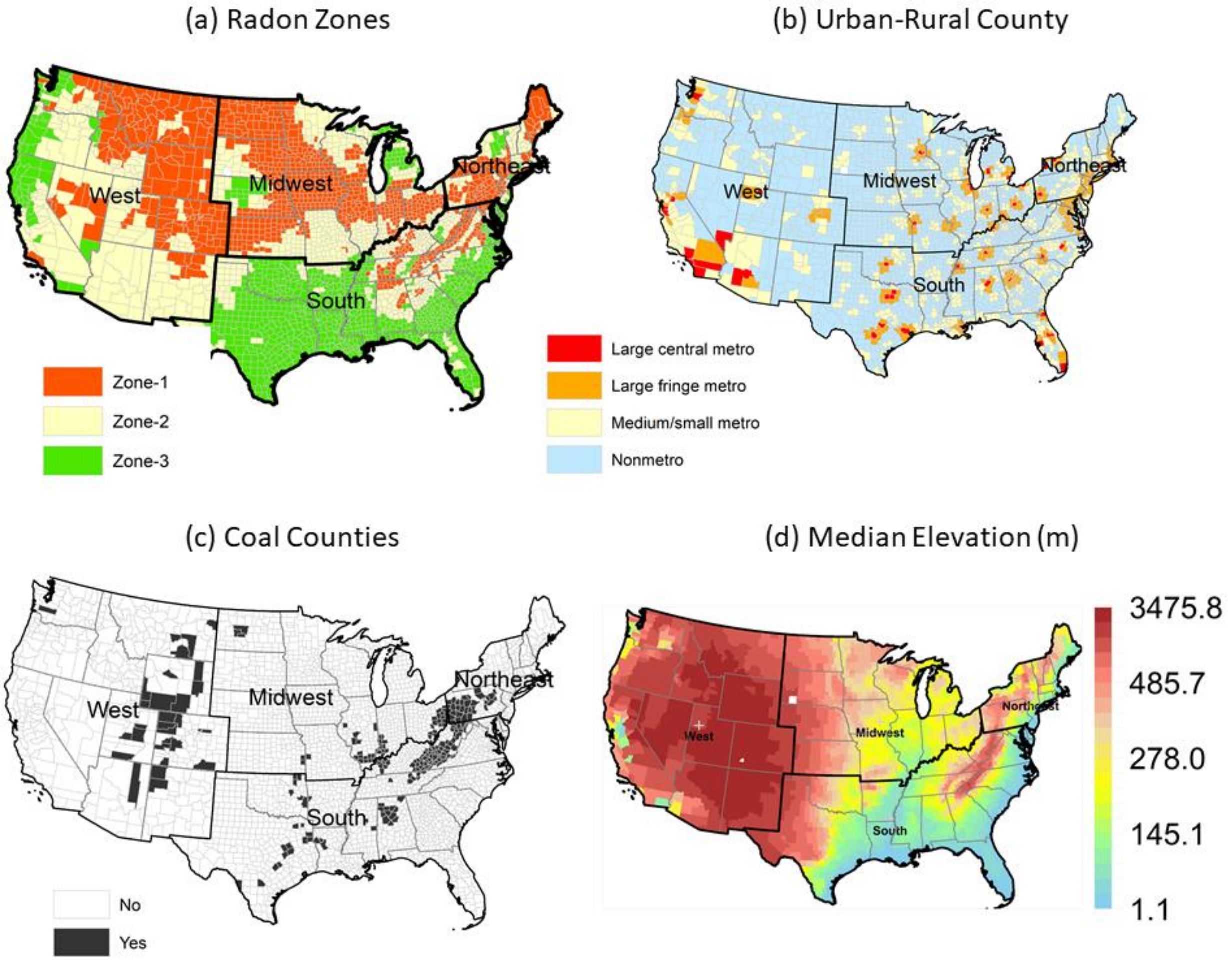

Figure S5. (a) Spatial distribution, training, validation, and test counties, and (b) distribution of LBC mortality rate in all data, training, validation, and test data. Map in Figure S5a was created in the R (version 4.1.1) Statistical Computing Environment [1].

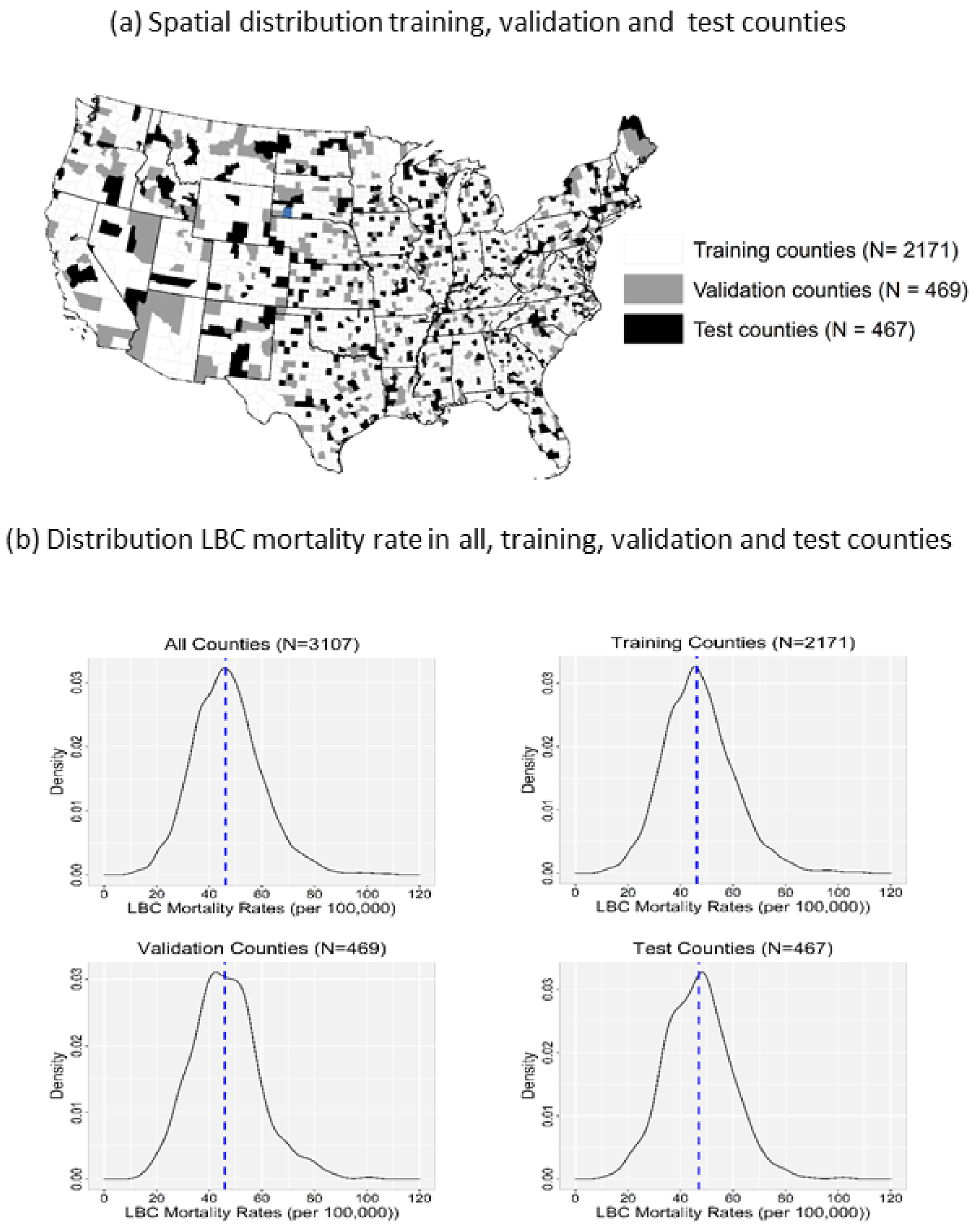

Figure S6. Pearson correlation matrix plots. In this plot, correlation coefficients are colored according to the value. The correlation matrix can also be reordered according to the degree of association between variables. Positive correlations are displayed in blue and negative correlations in red color. The color intensity and the size of the circle are proportional to the correlation coefficients. On the right side of the correlogram, the legend color shows the correlation coefficients and the corresponding colors. The correlations with p-value > 0.05 are considered as insignificant. In this case, the correlation coefficient values are left blank.

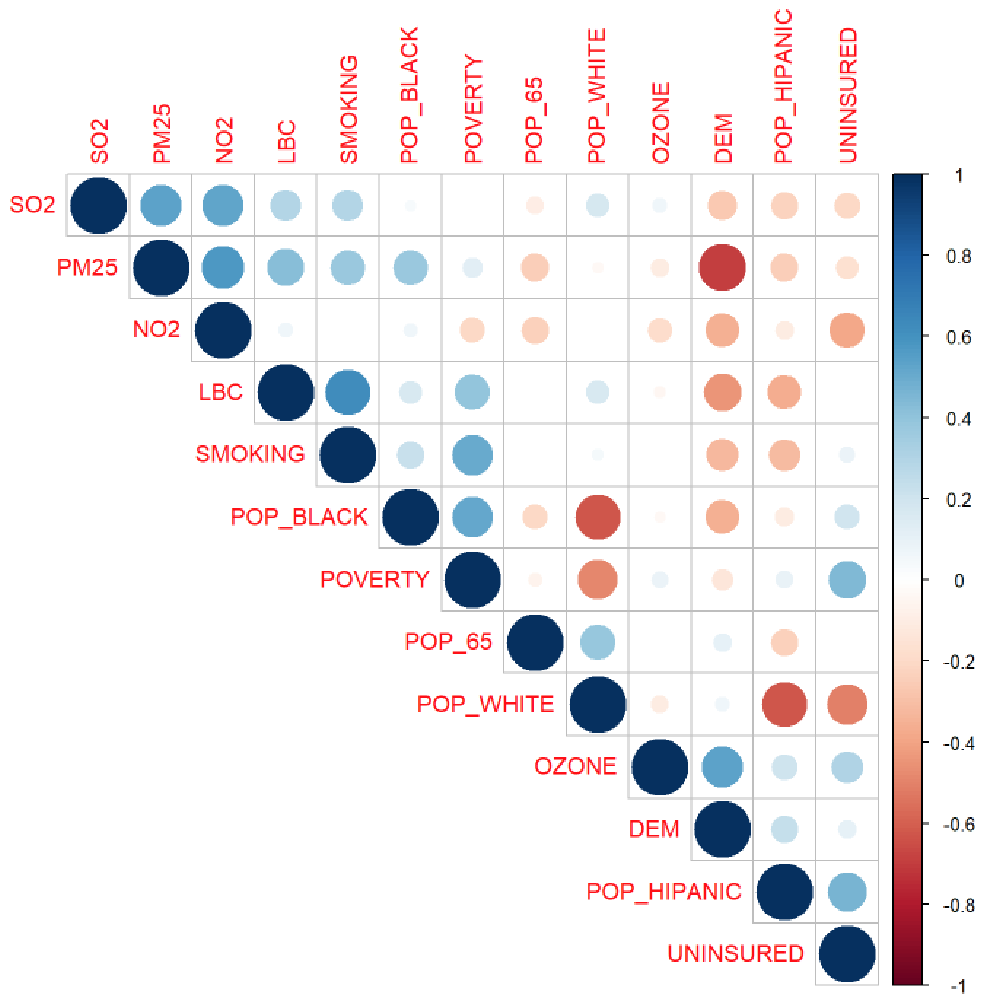

Figure S7. Mean LBC mortality rates in relation to the (a) urban-rural area; (b) radon-sones and (c) coal productions.

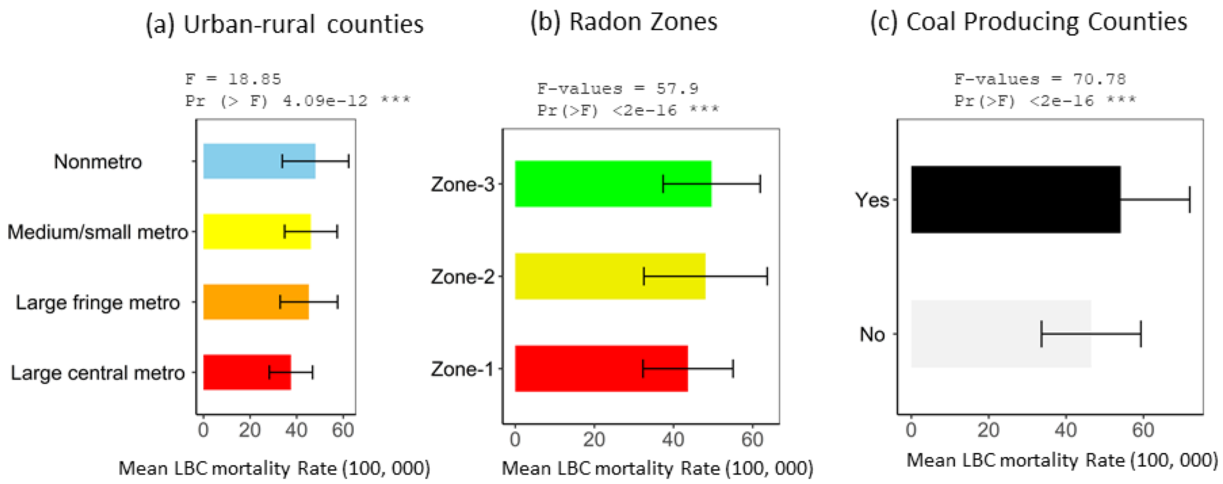

Figure S8. Global bivariate Moran’s I

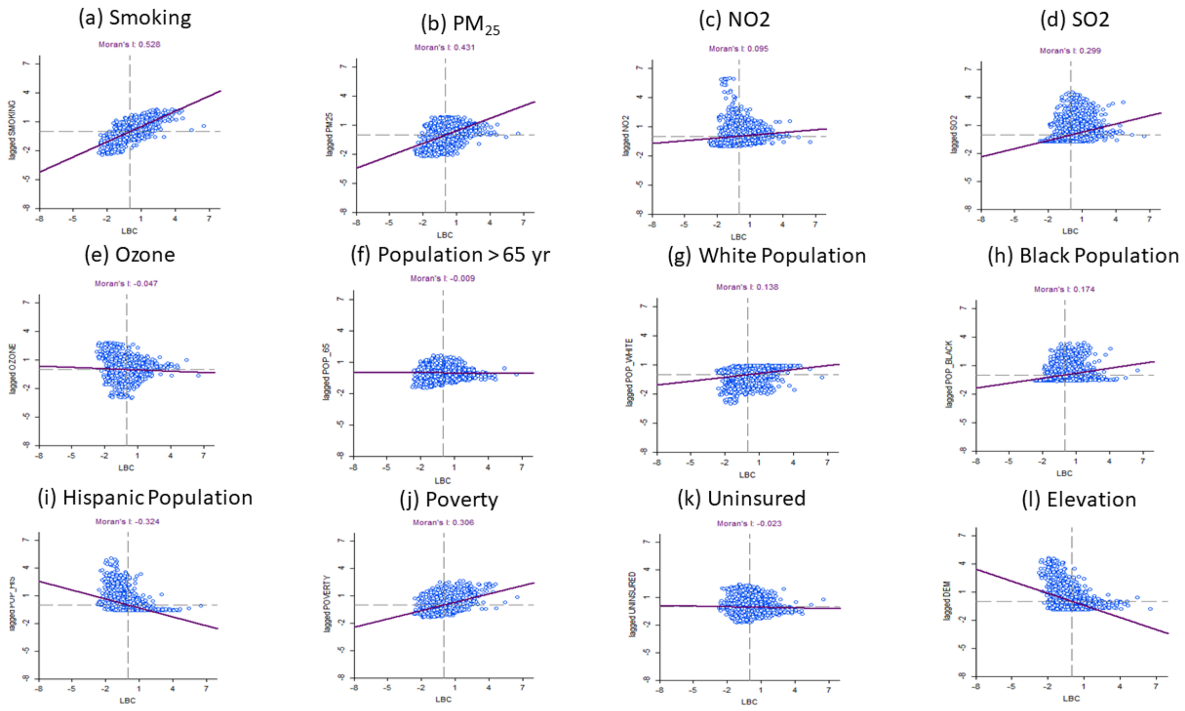

Figure S9. Bivariate LMI cluster of LBC mortality rates and twelve risk factors. Analysis and maps were generated in GeoDa (version 1.14), an open source software for geodata analysis [2].

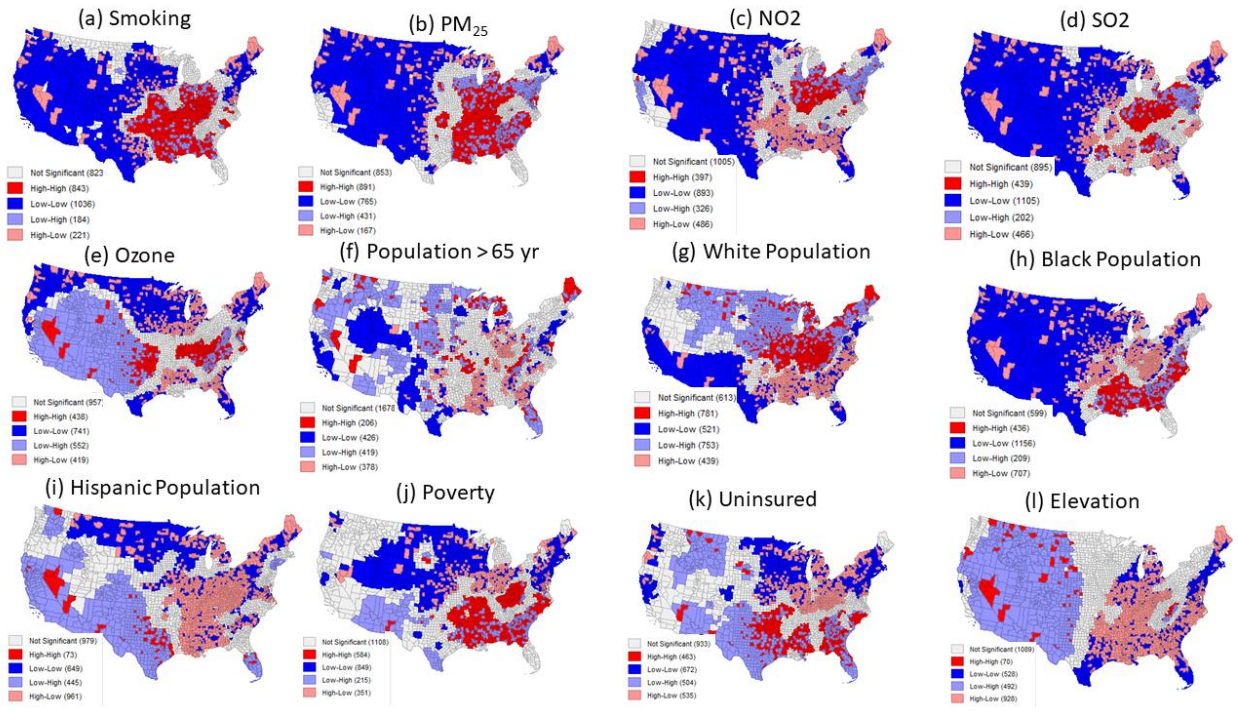

Figure S10. 1:1 plot of observed versus predicted LBC mortality rate in 667 test counties. All models were trained and validated with data from 2171 and 468 counties, respectively.

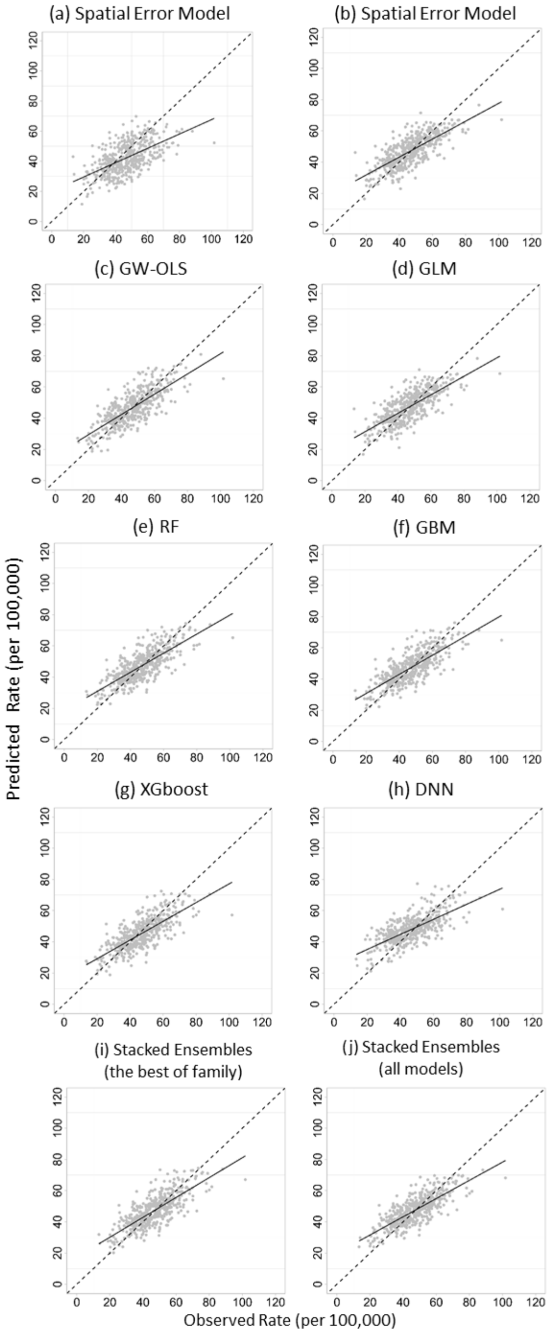

Figure S11. Bias^2 and variance of spatial regression and machine learning models

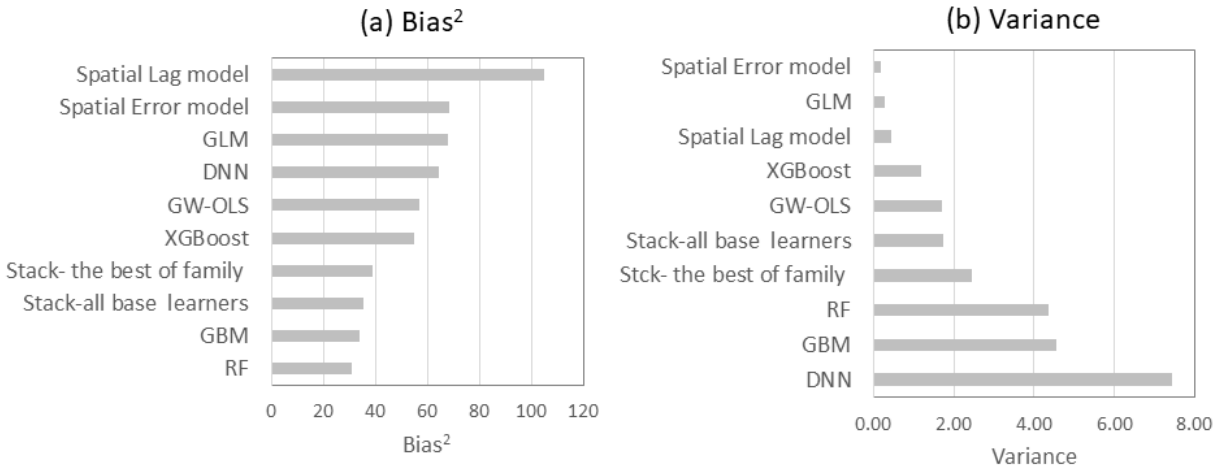

Table S1. Data source and years LBC mortality rates and risk-factors

Data	Years	Sources
Lung and Bronchus Cancer (LBC) Mortality Rates by County	2013-2017	https://statecancerprofiles.cancer.gov/
Cigarette Smoking Prevalence	2008-2017	Dwyer -Lindgren et al. (2014) [3]; County Health Ranking [4]
Poverty Rate	2012-2016	
Uninsured	2013-2017	County Health Ranking [4]
Demography	2013-2017	Census Population Estimates, http://www.census.gov
Black or African American, non-Hispanic population (%)		
White, non-Hispanic population (%)		
Hispanic/Latino Population (%)		
Population Aged 65 and Older (%)		
Air pollution		
$PM_{2.5}$	2006-2016	https://catalog.data.gov/dataset/daily-pm2-5-concentrations-all-county-2001-2016
NO_2	2003-2012	http://fizz.phys.dal.ca/~atmos/martin/?page_id=232 [5]
SO_2	2005-2015	
Ozone	2007-2016	https://data.cdc.gov/api/views/kmf5-t9yc/rows.csv?accessType=DOWNLOAD
Coal production	2006-2016	https://www.eia.gov/coal/data.php#production
Urban-Rural area	2016	https://www.cdc.gov/nchs/data_access/urban_rural.htm#2006_Urban-Rural_Classification_Scheme_for_Counties
Elevation		http://www.usgs.gov)
Randon-zone		https://www.epa.gov/radon/epa-map-radon-zones

Table S2. Summary statistics of mean LBC mortality rates and risk factors of all, training, validation, and test counties.

Variables	N	mean	median	min	max	q25	q75	sd	skew
	All Data								
LBC mortality rates	3107	47.05	46.30	10.10	134.70	37.90	54.80	13.43	0.67
Smoking	3107	21.42	21.20	8.70	33.60	19.20	23.70	3.28	0.04
PM_{25}	3107	7.12	7.43	2.48	11.03	5.81	8.44	1.77	-0.43
NO_2	3107	1.29	0.99	0.15	13.21	0.62	1.66	1.06	3.36
SO_2	3107	0.03	0.01	0.00	0.25	0.00	0.03	0.04	2.50
Ozone	3107	39.60	39.62	28.04	51.52	37.74	41.15	2.84	0.21
Population > 65 years	3107	18.43	18.10	3.80	55.60	15.50	20.80	4.54	0.80
White population	3107	77.20	84.46	3.02	98.18	65.52	93.00	19.60	-1.20
Black population	3107	9.02	2.20	0.00	84.92	0.66	10.31	14.34	2.27
Hispanic population	3107	9.20	3.98	0.44	95.98	2.12	9.40	13.64	3.09
Poverty	3107	16.70	15.84	3.26	46.14	12.04	20.08	6.34	0.98
Uuinsured	3107	13.16	12.58	2.42	33.82	9.45	16.17	4.90	0.72
Elevation	3107	441.65	278.03	1.12	3475.76	145.09	485.68	507.72	2.42
	Training Data								
LBC mortality rates	2171	47.21	46.20	10.10	134.70	37.82	55.00	13.64	0.70
Smoking	2171	21.45	21.30	8.70	33.60	19.20	23.80	3.33	0.01
PM_{25}	2171	7.12	7.43	2.48	11.03	5.81	8.43	1.77	-0.41
NO_2	2171	1.29	0.98	0.17	13.21	0.63	1.66	1.08	3.54
SO_2	2171	0.03	0.01	0.00	0.24	0.00	0.03	0.04	2.40
Ozone	2171	39.57	39.64	28.04	51.52	37.72	41.15	2.86	0.17
Population > 65 years	2171	18.43	18.10	3.80	55.60	15.60	20.90	4.58	0.82
White population	2171	76.84	84.28	3.62	98.18	64.91	92.97	19.93	-1.19
Black population	2171	9.15	2.26	0.00	84.92	0.64	10.73	14.46	2.25
Hispanic population	2171	9.27	3.98	0.44	95.98	2.12	9.54	13.86	3.12
Poverty	2171	16.87	15.92	3.26	45.44	12.08	20.43	6.44	0.95
Uuinsured	2171	13.20	12.62	2.42	32.74	9.48	16.14	4.92	0.74
Elevation	2171	442.55	284.30	1.76	3475.76	147.48	486.54	505.15	2.44
	Validation Data								
LBC mortality rates	469	46.56	46.00	16.00	120.30	37.89	53.80	13.44	0.79
Smoking	469	21.29	21.10	12.90	30.80	19.10	23.50	3.14	0.08
PM_{25}	469	7.04	7.40	2.78	10.73	5.73	8.40	1.76	-0.42
NO_2	469	1.25	0.96	0.15	8.55	0.60	1.60	0.97	2.64
SO_2	469	0.02	0.01	0.00	0.21	0.00	0.03	0.03	2.47
Ozone	469	39.69	39.59	29.28	50.59	37.90	41.20	2.92	0.28

Variables	N	mean	median	min	max	q25	q75	sd	skew
Population > 65 years	469	18.46	18.20	9.00	37.90	15.40	20.70	4.26	0.75
White population	469	77.76	84.52	3.02	97.68	65.88	93.42	19.47	-1.19
Black population	469	8.48	1.86	0.04	72.78	0.68	9.10	13.72	2.22
Hispanic population	469	9.68	4.04	0.56	95.12	2.00	8.66	14.98	2.93
Poverty	469	16.29	15.50	4.58	46.14	12.12	19.44	6.04	1.07
Uuinsured	469	13.07	12.52	3.32	29.70	9.50	16.06	4.80	0.62
Elevation	469	456.24	285.85	1.12	3330.83	146.54	521.73	525.06	2.44
	Test Data								
LBC mortality rates	467	46.83	47.00	13.39	101.80	38.09	54.90	12.42	0.33
Smoking	467	21.41	21.20	12.50	33.30	19.00	23.70	3.17	0.16
PM_{25}	467	7.17	7.51	2.54	10.66	5.94	8.53	1.76	-0.52
NO_2	467	1.33	1.06	0.19	9.70	0.62	1.77	1.06	2.91
SO_2	467	0.03	0.01	0.00	0.25	0.00	0.03	0.04	2.70
Ozone	467	39.65	39.62	30.48	48.40	37.69	41.11	2.71	0.33
Population > 65 years	467	18.43	17.90	6.40	38.30	15.40	20.70	4.60	0.78
White population	467	78.27	84.64	9.58	97.86	67.73	92.71	18.15	-1.21
Black population	467	8.93	2.26	0.04	76.42	0.65	10.24	14.41	2.37
Hispanic population	467	8.38	4.02	0.58	63.48	2.32	9.16	10.89	2.65
Poverty	467	16.37	15.46	4.90	44.74	11.92	19.68	6.16	1.03
Uuinsured	467	13.07	12.38	3.36	33.82	9.27	16.34	4.91	0.69
Elevation	467	422.81	255.43	2.12	2755.99	136.76	454.73	502.40	2.27

Table S3. List of hyper-parameters used to conduct the grid-search and to select the best parameters for the generalized linear model (GLM), distributed random forest (DRF), extreme gradient boosting machine (XGBoost), and Deep Neural Network (DNN).

Algorithms	Tuning parameters		Best parameters
GLM	alpha	0-1 by 0.25	0
	lambda	0-1, by 0.1	1
XGBoost	ntrees	50-500 by 50	350
	max_depth	1-20, by 1	4
	min_rows	1, 5, 20, 50, and 100	50
	learn_rate	0.001-0.01, by 0.001	0.01
	sample_rate	0.3-1.0, by 0.05	0.75
	col_sample_rate	0.3-1.0, by 0.05	0.85
	col_sample_rate_per_tree	0.3-1.0, by 0.05	0.8
RF	ntrees	50- 5000, by = 50	576
	max_depth	10, 20, 30, and 40	30
	sample_rate	0-1, by=0.2	0.6
DNN	activation	Rectifier, Maxout, Tanh, RectifierWithDropout	Tanh
		MaxoutWithDropout, and TanhWithDropout	
	epochs	5, 200, by 5	75
	hidden	(5, 5, 5, 5, 5), (10, 10, 10, 10), (50, 50, 50), and c(100, 100, 100)	c(100, 100, 100)
	rate	0, 01, 0.005, and 0.001	0.001
	rate_annealing	1e-8, 1e-7, and 1e-6	1.00E-08
	rho	0.9, 0.95, 0.99, and 0.999	0.99
	epsilon	(1e-10, 1e-8, 1e-6, and 1e-4	1.00E-08
	l1	0, 0.0001, and 0.00001	1.00E-08
	l2	0, 0.0001, and 0.00001	1.00E-04
	momentum_start	0 and 0.5	0
	momentum_stable	0, 0.5, and 0.99	0.00E+00
	input_dropout_ratio	0, 0.1, and 0.2	0.01
	max_w2	10, 100, 1000, and 3.4028235e+38	100
GBM	`col_sample_rate`	`seq(0.3,1,0.05)`	0.35
	`col_sample_rate_per_tree`	`seq(0.3,1,0.05)`	0.95

Algorithms	Tuning parameters		Best parameters
	learn_rate	seq(0.001,0.01,0.001)	0.0090
	max_depth	seq(1,20)	20
	min_rows	c(1,5,10,20,50,100)	1.0
	min_split_improvement	{1e-4, 1e-5}	
	ntrees	c(100,500, 1000)	500
	sample_rate	seq(0.3,1,0.05)	0.40

Table S4. Summary results of ordinary least square (OLS), spatial lag , spatial error geographically weighted ordinary least squares regression (GW-OLS) models.

	**OLS**	**Spatial lag**	**Spatial Error**	**GW-OLS**		
				**Min**	**Medium**	**Max**
Intercept	47.30***	35.24***	47.28***	38.15	46.32	57.39
Smoking	3.88***	3.027***	3.18***	-0.03	2.14	4.43
PM_{25}	0.22	-0.22	0.50	-10.74	0.58	6.64
NO_2	-0.06	0.14	-0.32	-8.11	-0.29	4.85
SO_2	0.33	0.20	0.633*	-11.37	0.09	7.45
Ozone	2.33***	1.911***	2.207***	-4.61	2.62	9.78
Population > 65 years	-0.979***	-0.731***	-0.842***	-3.42	-0.23	4.01
White population	4.531***	3.395***	4.085***	-3.23	4.45	45.22
Black population	-0.68	-0.69	-0.80	-7.66	2.72	29.34
Hispanic population	-0.902**	-0.732*	-1.156**	-4.89	0.55	41.44
Poverty	4.625***	3.773***	4.486***	-0.81	4.01	9.53
Uuinsured	-0.48	-0.463*	-0.29	-4.89	-0.29	2.43
Elevation	-4.90***	-3.99**	-4.98***	-24.72	-8.00	1.65
Coal production(No)	-0.96	-0.52	-0.89	-12.30	-0.97	8.54
Radon zone 1	2.78***	2.11***	2.53***	-5.62	1.14	11.05
Radon zone 2	1.271**	0.880*	1.462***	-5.78	1.07	11.03
Large central metro	-2.27	-2.21	-2.27	-11.77	-2.53	11.25
Large fringe metro	-0.31	-0.08	-0.65	-7.48	0.42	7.44
Medium/small metro	-1.898***	-1.798***	-1.945***	-4.56	-1.30	2.04
Observations	2,171	2,171	2,171		2,171	
R^2	0.58				0.69	
Adjusted R^2	0.50				0.65	
Log Likelihood		-7,728.55	-7,748.43			
sigma2		71.136	72.146			
Akaike Inf. Crt		15,499.10	15,538.85		15380	
Residual Std. Error	8.83 (df =2152)					
F-Statistic	167.86*** (df=18; 2152)					
Wald Test (df =1)		147.35***	119.29***			
LR Test (df =1)		143.62***	103.87***			
Rho		0.255				
Lambda			0.283			
Kernel function					Bisquare	
Adaptive bandwidth					453	

Additional Text for the Methods:

Text1: Hot Spot Analysis (Getis-Ord Gi*)

The Hot Spot Analysis calculates the Getis-Ord Gi* statistic (pronounced G-i-star) for LBC mortality rates [6]. The resultant -Ord Gi* statistics (*z*-score and *p*-values) indicate areas with either high or low-value clusters. Larger z-scores (statistically significant positive z-scores) show more intense clustering of high values, and smaller z-score (statistically significant negative *z*-scores), suggest more intense clustering of low values.

The Getis-Ord local statistic is given as:

$$G_i^* = \frac{\sum_{j=1}^{n} w_{i,j} x_j - \bar{X} \sum_{j=1}^{n} w_{i,j}}{S \sqrt{\frac{\left[n \sum_{j=1}^{n} w_{i,j}^2 - \left(\sum_{j=1}^{n} w_{i,j}\right)^2\right]}{n-1}}} \quad [1]$$

where x_j is the attribute value for feature j, $w_{i,j}$ is the spatial weight between feature i and j, n is equal to the total number of features.

Text 2: Bivariate Moran-I

Bivariate Moran-I describes the relationship between the value for one variable at location *i*, *xi*, and the average of the neighboring values for *another variable*, i.e., its spatial lag ∑*jwijyj*. This statistic is the product of *xi* with the spatial lag of *yi* (i.e., ∑*jwijyj*), with both variables standardized, such that their means are zero and variances equal one:

$$I_i^B = c x_i \sum_j w_{ij} y_j, \quad \textbf{[2]}$$

where *wij* are the elements of the spatial weights matrix.

We used GeoDa (1.14) for Bivariate Moran-I analysis [2]

Text 3: Spatial stratified heterogeneity (SSH)

Spatial stratified heterogeneity (SSH) is one of the basic features of spatial data, representing a variation of an attribute value between strata or areas with measures by q-statistics [7]:

$$q = 1 - \frac{\sum_{h=1}^{L}\sum_{i=1}^{N_h}(Y_{hi} - \bar{Y}_h)^2}{\sum_{i=1}^{N}(Y_i - \bar{Y})^2} = 1 - \frac{\sum_{h=1}^{L} N_h \sigma_h^2}{N\sigma^2} = 1 - \frac{SSW}{SST}$$ [3]

.

Text 4: Spatial Lag Models

Spatial lag models are extensions of regression models which allow observations of the dependent variable y in area i $(i = 1, \ldots, n)$ to depend on observations in neighboring areas $j \neq i$ [8]. The basic spatial lag model, the so-called first-order spatial autoregressive (SAR) model, takes the form:

$$y_i = \rho \sum_{j=1}^{n} W_{ij} y_j + \sum_{q=1}^{Q} X_{iq} \beta_q + \varepsilon_i$$ **[4]**

where the error term, ei, is iid. Wijis the (i, j) th element of the n-by-n spatial weights matrix W. The scalar q in Eq. [3] is a parameter (to be estimated) that will determine the strength of the spatial autoregressive relation between yi and RjWij yj; a linear combination of spatially related observations based on non-zero elements in the ith row of W.

Text 5: Spatial Error Models

Another form of spatial dependence occurs when the dependence works through the error process, in that the errors from different areas may display spatial covariance [8]. The most common specification is a spatial autoregressive process of the first order, as given by

$$\varepsilon_i = \lambda \sum_{j=1}^{n} W_{ij}\varepsilon_j + u_i$$ **[5]**

The spatial error model (SEM) may be viewed as a combination of a standard regression model with a spatial autoregressive model in the error term e, and hence has an expectation equal to that of the standard regression model.

Text 6: Geographically Weighted OLS Regression (GW-OLS).

The GW-OLS involves spatial regression techniques increasingly used when data are not described well by a global model [17]. GW-OLS explores spatial heterogeneity in the relationships between variables where non-stationarity exists such that locally weighted regression coefficients move away from their global values. GW-OLS fits a regression equation for every location in the dataset, incorporating the dependent and explanatory variables falling within the user-selected bandwidth of each target location. The bandwidth's shape and size usually depend on the kernel type, bandwidth method, distance, and the number of neighbors parameters. We used "bi-square kernels" with adaptive distance to select the optimum neighbor size.

Text 7: Base Machine Learning Models

Text 7.1. Generalized Linear Model (GLM)

The GLM is a flexible generalization of ordinary linear regression that allows for response variables with error distribution models other than normal [9]. The GLM generalizes linear regression by allowing the linear model to be related to the response variable via a link function.

Text 7.2: Random Forest (RF)

Random forests [10] is a modification of bagging or bootstrap aggregation, that builds a large collection of de-correlated trees and then averages them for reducing the variance of an estimated prediction function. A bootstrap sample is chosen at random with replacement from the data. Some observations end up in the bootstrap sample more than once, while others are not included ("out of bag", OOB). The excluded OOB data are predicted from the bootstrap samples and by combining the OOB predictions from all trees. Unlike linear regression, RF has no requirements concerning the form of the probability density function of the dependent variable [11]. RF allows estimation of the variable importance by calculating percentage increase in mean square error by shuffling the values of the OOB samples.

Text 7.3: Gradient boosting machine (GBM)

The Gradient boosting machine *(*GBM) model used a tree-based ensemble of weak models (decision trees) and was initially introduced by Friedman in 2011 [12] for both classification and regression problems. Whereas RF builds an ensemble of deep independent trees, GBMs build an ensemble of shallow trees (weak learner or regression tree) in sequence with each tree learning and improving on the previous one. A loss or cost function (how good are model's coefficients are at fitting the underlying data), a weak learner (regression tree) and an additive model (which add weak learners or trees to minimize the loss function) are the three fundamental components of GBM. A gradient descent procedure is used to minimize the loss when adding trees. The general idea of gradient descent is to tweak parameter(s) iteratively to minimize a cost function, the error between predicted values, and the actual values.

Text 7.4: Extreme Gradient boosting (XGBoost)

Extreme Gradient boosting (XGBoost) is an optimized distributed Gradient boosting library that is designed to be efficient, flexible, and portable across multiple languages [13,14]. Like GBM, it implements an ensemble learning technique by building many models sequentially, with each new model attempts to correct the error of the previous tree model. However, XGBoost has a few advantages over traditional boosting, such as *parallel preprocessing* (at the node level), which makes it faster than GBM, *regularization* techniques that reduce overfitting and improve overall performance, and *early stopping* so that we can stop model assessment when additional trees offer no improvement.

Text 7.5. Deep Neural Network (DNN)

The *Deep Neural Network (*DNN) is based on a multi-layer, feed-forward (artificial neural network (ANN) that is trained with stochastic gradient descent (iterative method for optimizing loss function) by updating weight using back-propagation (fine-tuning the weights of a neural net based on the error rate (i.e. loss) obtained in the previous epoch or iteration). The network may contain many hidden layers consisting of nodes, and there may be intermediate layers between the input and output layers. Each neuron is characterized by its weight, bias, and activation function. A non-linear activation or transfer function is applied to activate a neuron by calculating the weighted sum of inputs and further adding bias to it. The output from the activation function moves to the next hidden layer, and the same process is repeated. This forward movement of information is known as *forward propagation.* Based on this error value calculated from *forward propagation*, the neurons' weights and biases are updated, known as s *back-propagation*. Advanced features such as adaptive learning rate, rate

annealing, momentum training, dropout, L1 or L2 regularization, check-pointing, and grid search enable the DNN to achieve high predictive accuracy.

Text 8: Stack-Ensemble Model

Ensemble machine learning with stack generalization uses multiple learning algorithms to obtain better predictive performance than could be obtained from any of the constituent learning algorithms. Many of the popular modern machine learning algorithms are ensembles. For example, Random Forest and Gradient Boosting Machine are both ensemble learners. However, stacked generalization or stacking or supper learning [15], that introduces the concept of a meta-learner that ensembles or combines several strong, weak, and diverse sets of machine learning models (level-0 base models) to get better prediction [16]. In this modeling approach, each base-level model is trained first, then the meta-model (level-1 generalizer) is trained on the outputs of the base-level models. The base-level models often consist of different learning algorithms, and therefore stacking ensembles are often heterogeneous.

The steps below describe the individual tasks involved in training and testing a Super Learner ensemble. H_2O automates most of the steps below so that you can quickly and easily build ensembles of H_2O models.

1. Set up the ensemble.
    a. Specify a list of L base algorithms (with a specific set of model parameters).
    b. Specify a metalearning algorithm.
2. Train the ensemble.
    a. Train each of the L base algorithms on the training set.
    b. Perform k-fold cross-validation on each of these learners and collect the cross-validated predicted values from each of the L algorithms.
    c. The N cross-validated predicted values from each of the L algorithms can be combined to form a new N x L matrix. This matrix, along with the original response vector, is called the “level-one” data. (N = number of rows in the training set.)

d. Train the metalearning algorithm on the level-one data. The "ensemble model" consists of the L base learning models and the metalearning model, which can then be used to generate predictions on a test set.

3. Predict on new data.

   a. To generate ensemble predictions, first generate predictions from the base learners.
   b. Feed those predictions into the metalearner to generate the ensemble prediction

References:

1 R-core-Team. R: A language and environment for statistical computing. R Foundation for Statistical Computing, Vienna, Austria. URL, https://www.R-project.org/. (2021).
2 Anselin, L., Syabri, I. & Kho, Y. in *Handbook of applied spatial analysis* 73-89 (Springer, 2010).
3 Dwyer-Lindgren, L. *et al.* US County-Level Trends in Mortality Rates for Major Causes of Death, 1980-2014US County-Level Trends in Mortality Rates for Major Causes of DeathUS County-Level Trends in Mortality Rates for Major Causes of Death. *JAMA* **316**, 2385-2401, doi:10.1001/jama.2016.13645 (2016).
4 Institute, U. o. W. P. H. *2020 Measures*, <https://www.countyhealthrankings.org/explore-health-rankings/measures-data-sources/2020-measures> (2020).
5 Geddes, J. A., Martin, R. V., Boys, B. L. & Donkelaar, A. v. Long-Term Trends Worldwide in Ambient NO2 Concentrations Inferred from Satellite Observations. *Environmental Health Perspectives* **124**, 281-289, doi:doi:10.1289/ehp.1409567 (2016).
6 Getis, A. & Ord, J. K. The Analysis of Spatial Association by Use of Distance Statistics. *Geographical Analysis* **24**, 189-206, doi:https://doi.org/10.1111/j.1538-4632.1992.tb00261.x (1992).
7 Wang, J.-F., Zhang, T.-L. & Fu, B.-J. A measure of spatial stratified heterogeneity. *Ecological Indicators* **67**, 250-256 (2016).
8 Haining, R. P. & Haining, R. *Spatial data analysis: theory and practice*. (Cambridge university press, 2003).
9 Breslow, N. E. Generalized linear models: checking assumptions and strengthening conclusions. *Statistica Applicata* **8**, 23-41 (1996).
10 Breiman, L. Random forests. *Machine Learning* **45**, 5-32, doi:Doi 10.1023/A:1010933404324 (2001).
11 Kuhn, M. & Johnson, K. *Applied predictive modeling*. Vol. 26 (Springer, 2013).
12 Friedman, J. H. Greedy function approximation: a gradient boosting machine. *Annals of statistics*, 1189-1232 (2001).
13 Chen, T. & He, T. Xgboost: extreme gradient boosting. (2019).
14 Boehmke, B. & Greenwell, B. M. *Hands-on machine learning with R*. (CRC Press, 2019).
15 Wolpert, D. H. Stacked generalization. *Neural Networks* **5**, 241-259, doi:https://doi.org/10.1016/S0893-6080(05)80023-1 (1992).

16 Saliba, R., Callieris, R., D’Agostino, D., Roma, R. & Scardigno, A. Stakeholders’ attitude towards the reuse of treated wastewater for irrigation in Mediterranean agriculture. *Agricultural Water Management* **204**, 60-68 (2018).
